# Conserved and lineage-specific hypothetical proteins may have played a central role in the rise and diversification of major archaeal groups

**DOI:** 10.1186/s12915-022-01348-6

**Published:** 2022-07-05

**Authors:** Raphaël Méheust, Cindy J. Castelle, Alexander L. Jaffe, Jillian F. Banfield

**Affiliations:** 1 Department of Earth and Planetary Science, University of California, Berkeley, CA USA; 2 Innovative Genomics Institute, University of California, Berkeley, CA USA; 3 LABGeM, Génomique Métabolique, Genoscope, Institut François Jacob, CEA, Evry, France; 4 Chan Zuckerberg Biohub, San Francisco, CA USA; 5 Department of Plant and Microbial Biology, University of California, Berkeley, CA USA; 6 Department of Environmental Science, Policy, and Management, University of California, Berkeley, CA USA

**Keywords:** Archaea, Protein family, Comparative genomics, Bioinformatics

## Abstract

**Background:**

Archaea play fundamental roles in the environment, for example by methane production and consumption, ammonia oxidation, protein degradation, carbon compound turnover, and sulfur compound transformations. Recent genomic analyses have profoundly reshaped our understanding of the distribution and functionalities of Archaea and their roles in eukaryotic evolution.

**Results:**

Here, 1179 representative genomes were selected from 3197 archaeal genomes. The representative genomes clustered based on the content of 10,866 newly defined archaeal protein families (that will serve as a community resource) recapitulates archaeal phylogeny. We identified the co-occurring proteins that distinguish the major lineages. Those with metabolic roles were consistent with experimental data. However, two families specific to Asgard were determined to be new eukaryotic signature proteins. Overall, the blocks of lineage-specific families are dominated by proteins that lack functional predictions.

**Conclusions:**

Given that these hypothetical proteins are near ubiquitous within major archaeal groups, we propose that they were important in the origin of most of the major archaeal lineages. Interestingly, although there were clearly phylum-specific co-occurring proteins, no such blocks of protein families were shared across superphyla, suggesting a burst-like origin of new lineages early in archaeal evolution.

**Supplementary Information:**

The online version contains supplementary material available at 10.1186/s12915-022-01348-6.

## Background

Until recently, the archaeal domain comprised only two phyla, the Euryarchaeota and the Crenarchaeota, most of which were described from extreme environments [1, 2]. The recovery of genomes from metagenomes without the prerequisite of laboratory cultivation has altered our view of diversity and function across the Archaea domain [3–5]. Hundreds of genomes from little studied and newly discovered archaeal clades have provided new insights into archaeal metabolism and evolution. Now, Archaea include at least four major large groups, the Euryarchaeota (cluster I and cluster II) [3–5], the TACK (the monophyletic group comprising the Thaumarchaeota, Aigarchaeota, Crenarchaeota, and Korarchaeota also known as Proteoarchaeota) [6], the Asgard [7, 8], and the DPANN (Diapherotrites, Parvarchaeota, Aenigmarchaeota, Nanoarchaeota, Nanohaloarchaea) [9, 10], all of which comprise several distinct phylum-level lineages. These archaea are not restricted to extreme habitats, but are widely distributed in diverse ecosystems [3–5].

Most studies have focused on the metabolic potential of archaea based on analysis of proteins with known functions and revealed roles in the carbon, nitrogen, hydrogen, and sulfur biogeochemical cycles. For example, Euryarchaeota includes many methanogens and non-methanogens, including heterotrophs and sulfur oxidizers [11]. The TACK includes Thaumarchaeota, most but not all of which oxidize ammonia [12–15], Aigarchaeota that tend to be chemolithotrophs that oxidize reduced sulfur compounds [16], Crenarchaeota that include thermophilic sulfur oxidizers [17], and Korarchaeota, a highly undersampled group represented by amino acid degraders, that anaerobically oxidize methane and also metabolize sulfur compounds [18]. The Asgard have variable metabolisms and their genomes encode pathways involved in structural components that are normally considered to be eukaryotic signatures [7, 8]. The DPANN are an intriguing group that typically has very small genomes and symbiotic lifestyles [19, 20]. Their geochemical roles are difficult to predict, given the predominance of hypothetical proteins. Previously, the distribution of protein families over bacterial genomes was used to provide a function rather than phylogeny-based clustering of lineages [21]. Protein clustering allows the comparison of the gene content between genomes by converting amino acid sequences into units of a common language. The method is agnostic and unbiased by preconceptions about the importance or functions of genes.

Here, we adapted this approach to evaluate the protein family-based coherence of the archaea and to test the extent to which a subdivision of archaea could be resolved based on shared protein family content. The analysis drew upon the large genome dataset that is now available for cultivated as well as uncultivated archaea (3197 genomes). The observation that hypothetical proteins (i.e., proteins lacking predicted functions) dominate the sets of co-occurring protein families that distinguish major archaeal groups indicates the importance of these protein sets in the rise of the major archaeal lineages.

## Results

### Genome reconstruction and collection

We collected 2618 genomes spanning all the recognized phyla and superphyla of the Archaea domain from the NCBI genome database (Additional file 1: Table S1). To enable our analyses, we augmented the relatively limited sampling of the DPANN by adding 569 newly available DPANN metagenome-assembled genomes (MAGs) from low oxygen marine ecosystems, an aquifer adjacent to the Colorado River, Rifle, Colorado, and from groundwater collected at the Genasci dairy farm, Modesto, California [22, 23]. The 3197 genomes were clustered at ≥ 95% average nucleotide identity (ANI) to generate 1749 clusters. We removed genomes with <70% completeness or >10% contamination or if there was < 50% of the expected columns in the alignment of 14 concatenated ribosomal proteins (see the “Methods” section). To avoid contamination due to mis-binning, we required that these proteins were co-encoded on a single scaffold. The average completeness of the final set of 1179 representative genomes is 95% and 928 were >90% complete (Additional file 1: Table S1). The 1179 representative genomes comprise 39 phylum-level lineages including 16 phyla that have more than 10 genomes (Additional file 1: Table S1 and Additional file 2: Fig. S1).

### Genomic content of representative genomes correlates with the phylogeny of archaea

We clustered the 2,336,157 protein sequences from the representative genomes in a two-step procedure to generate groups of homologous proteins (Additional file 2: Fig. S2). This resulted in 10,866 clusters (representing 2,075,863 sequences) that were present in at least five distinct genomes. These clusters are henceforth referred to as protein families.

We assessed the quality of the protein clustering. The rationale was that we expected protein sequences with the same function to cluster into the same protein family. We annotated our protein dataset using the Kyoto Encyclopedia of Genes and Genomes (KEGG) annotations [24] and systematically verified that the protein family groupings approximate functional annotations. The KEGG annotations in our dataset encompass 6482 unique annotations of various biological processes, including fast-evolving defense mechanisms. For each of these 6482 annotations, we reported the family that contains the highest percentage of protein members annotated with that KEGG annotation. Most clusters were of good quality. For 87% of the KEGG annotations (5627 out of 6482), one family always contained >80% of the proteins (Additional file 2: Fig. S3A). The contamination of each protein family was assessed by computing the percentage of the proteins with KEGG annotations that differ from the dominant annotation (percentage annotation admixture). Most of the families contain only proteins with the same annotation, and 2654 out of 3746 families (71%) have <20% annotation admixture (Additional file 2: Fig. S3B). Although this metric is useful, we note that it is imperfect because two homologous proteins can have different KEGG annotations and thus cluster into the same protein family, increasing the apparent percentage of annotation admixture. Although we used sensitive Hidden Markov Model-based (HMM-based) sequence-comparison methods and assessed the quality of the protein clustering, we cannot completely rule out the possibility that our pipeline failed to retrieve distant homology for highly divergent proteins. Small proteins and fast-evolving proteins are more likely to be affected. This lack of sensitivity would result in the separation of homologous proteins into distinct families and would impact the results. To reduce the incidence of proteins without functional predictions for which annotations should have been achieved we augmented PFAM and KEGG-based annotations by comparing sequences to the Protein Data Bank (PDB) database [25] and by performing HMM-HMM comparison against the eggNog database [26] (see the “Methods” section).

We compared our set of families to previous studies defining protein families of the archaeal domain, including the archaeal Clusters of Orthologous Genes (arCOGs) [27], and the functional phylogenomics consistent genome annotation UniFam [28]. Unifam used a centroid-based clustering on the protein sequences of 14,727 prokaryotic genomes comprising 360 archaeal genomes [28] whereas arCOGs used a bidirectional best hit approach on 168 archaeal genomes [27, 29]. We searched the arCOG and Unifam HMMs in the 2,336,157 protein sequences and detected HMM hits in 1,928,049 distinct sequences (83%). These comprise 1,890,925 sequences that group with 6584 out of 10,866 families (61%) and 37,124 sequences that did not cluster with any of the 10,866 families (Additional file 2: Fig. S4). More sequences were annotated using the arCOG (1,912,173) than the Unifam HMMs (1,376,811). Of note, we did not detect any hits with arCOG and Unifam HMMs for 184,938 sequences in the 10,866 families. Whereas arCOG and Unifam comprise 6584 families, our study identified 4282 new protein families. Out of the 4282 families, 157 families have a KEGG or a PFAM annotation and comprise phage and CRISPR-associated proteins, proteins with domain of unknown function and carbohydrate enzymes (Additional file 1: Table S3).

**Fig. 1:**
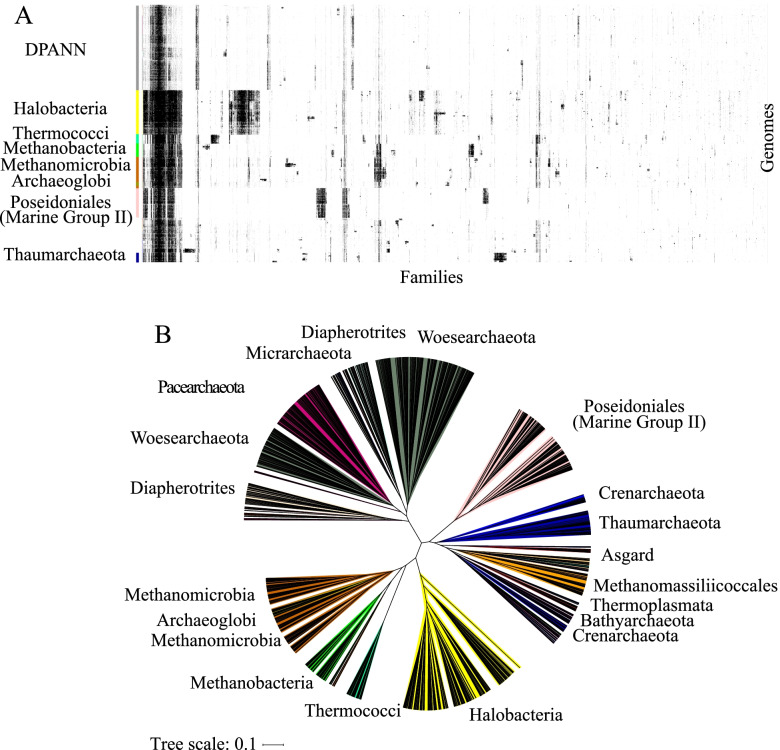
The distribution of the 10,866 families across the 1179 representative genomes. **A** The distribution of 10,866 widely distributed protein families (columns) in 1179 representative genomes (rows) from Archaea. Data are clustered based on the presence (black) and absence (white) profiles (Jaccard distance, complete linkage). **B** Tree resulting from the hierarchical clustering of the genomes based on the distributions of protein families in **A**

We visualized the distribution of the families over the genomes by constructing an array of the 1179 representative genomes (rows) vs. 10,866 protein families (columns) and hierarchically clustered the genomes based on profiles of protein family presence/absence (Fig. 1A). The families were also hierarchically clustered based on profiles of genome presence/absence. As previously reported for bacteria [21, 30], the hierarchical clustering tree of the genomes resulting from the protein clustering (Fig. 1B) correlated with the maximum-likelihood phylogenetic tree based on the concatenation of the 14 ribosomal proteins (Additional file 2: Fig. S1) (the cophenetic correlation based on a complete-linkage method is 0.83, based on average linkage 0.84, and based on single linkage, 0.84) (Additional file 2: Fig. S5). Although the tree resulting from the protein families correlates with the phylogenetic tree, it does not achieve the resolution of the phylogenetic tree, especially for placement of the deep branches. Interestingly, several phyla, such as the Crenarchaeota or the Woesarchaeota, are resolved into multiple groups (Fig. 1A). The first clade of Woesearchaeota corresponds to the Woesarchaeota-like I whereas the second clade groups together the Woesarchaeota and Woesarchaeota-like II groups. We could not evaluate the placement of Altiarchaeota relative to the DPANN because no genomes passed our quality control thresholds.

We defined modules as blocks of co-occurring protein families containing at least 20 families (see the “Methods” section) [21]. Each module was assigned a taxonomic distribution based on the taxonomy of the genomes with the highest number of families (see the “Methods” section and Additional file 1: Table S2). A block of 587 protein families that was broadly conserved across the 1179 genomes (left side in Fig. 1A) was designed as the module of “core families” (module 1) (Additional file 2: Fig. S6). Given their widespread distribution, it is unsurprising that most of the families are involved in well-known functions, including replication, transcription and translation and basic metabolism (oxidative phosphorylation chain, nucleotides, amino acids, ribosomal proteins, cofactors and vitamins, transporters, peptidases, DNA repair, and chaperones). As expected, many of these easily recognized core families, primarily those involved in energy metabolism and cofactor synthesis, are absent in DPANN genomes [9, 19] (Fig. 1A). Another interesting module (module 23) (Additional file 2: Fig. S6), composed of ~100 protein families, is widely distributed in most archaeal genomes but was not identified in DPANN and surprisingly, not in the Poseidoniales. Module 23 includes functions involved in carbon metabolism, amino-acid synthesis, and many transporter families. For instance, we identified several families for subunits of the Mrp antiporter as widespread in Halobacteria, Methanogens, and Thermococci, but they appear to be absent in DPANN and Poseidoniales. The Mrp antiporter functions as Na+/H+ antiporter and also contributes to sodium tolerance in Haloarchaea. Mrp has been reported to be involved in energy conservation in methanogens and in the metabolic system of hydrogen production in Thermococci.

The DPANN are an enigmatic set of lineages, the monophyly of which remains uncertain [31]. However, the protein family analysis clearly showed that these lineages group together and are distinct from other Archaea (Fig. 1B). A detailed protein family analysis of groups within the DPANN is presented elsewhere [22].

### Major clades possess groups of conserved protein families

We detected 96 modules that are restricted to non-DPANN lineages (Additional file 1: Table S2). Only 9 of the 96 modules were found in multiple phyla and in 8 of these 9 cases, the phyla that possess each module are phylogenetically unrelated (e.g., Crenarchaeota and Halobacteria). The 9th, module 44, is interesting in that it occurs in two phyla and those phyla are monophyletic (Thorarchaeota and Heimdallarchaeota of the Asgard superphylum). Thus, the vast majority of the non-DPANN modules (87) are restricted to a single phylum (Additional file 1: Table S2) and, perhaps surprisingly given phylogenetic support for superphyla within Archaea, almost no modules are specific to superphyla.

**Fig. 2:**
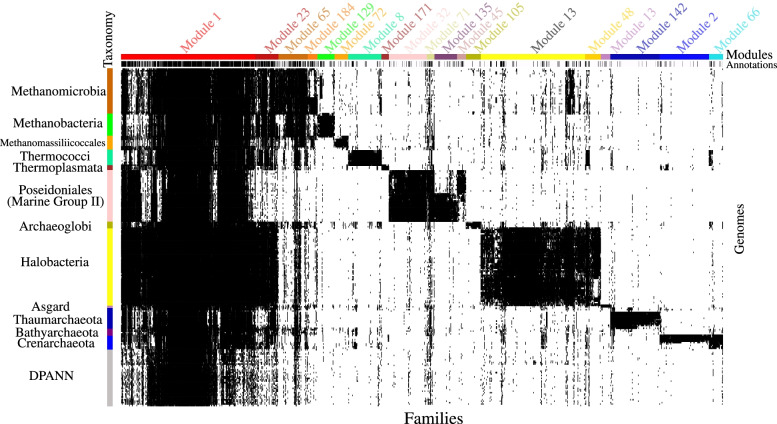
The distribution of the 2632 families of the 19 modules discussed in this study. Each column represents a protein family and each row represents a genome. Data are clustered based on the presence (black)/absence (white) profiles but also based on the taxonomy of the genomes and the module membership. The first colored top bar (annotations) shows the families with (black)/without (white) a predicted annotation whereas the second colored top bar (modules) indicates the module of each family. The colored side bar indicates the taxonomic assignment of each genome

Visualization of the distribution of protein families highlights the presence of 19 modules that are not only lineage specific but are also well conserved within each lineage (Fig. 2). In fact, we identified such archaeal group-specific modules in 10 out of 11 non-DPANN with more than 10 genomes (Additional file 2: Fig. S6 and Table 1). For instance, there are two modules (modules 13 and 108) comprising 525 families that are fairly conserved in Halobacteria. On average, each of the 525 families appears in 65% of the halobacterial genomes, yet these families are mostly absent in non-halobacterial genomes (Additional file 2: Fig. S7). These modules are slightly less conserved within each archaeal group than module 1 families (comprising core functions) (Additional file 2: Fig. S7).

**Table 1:**
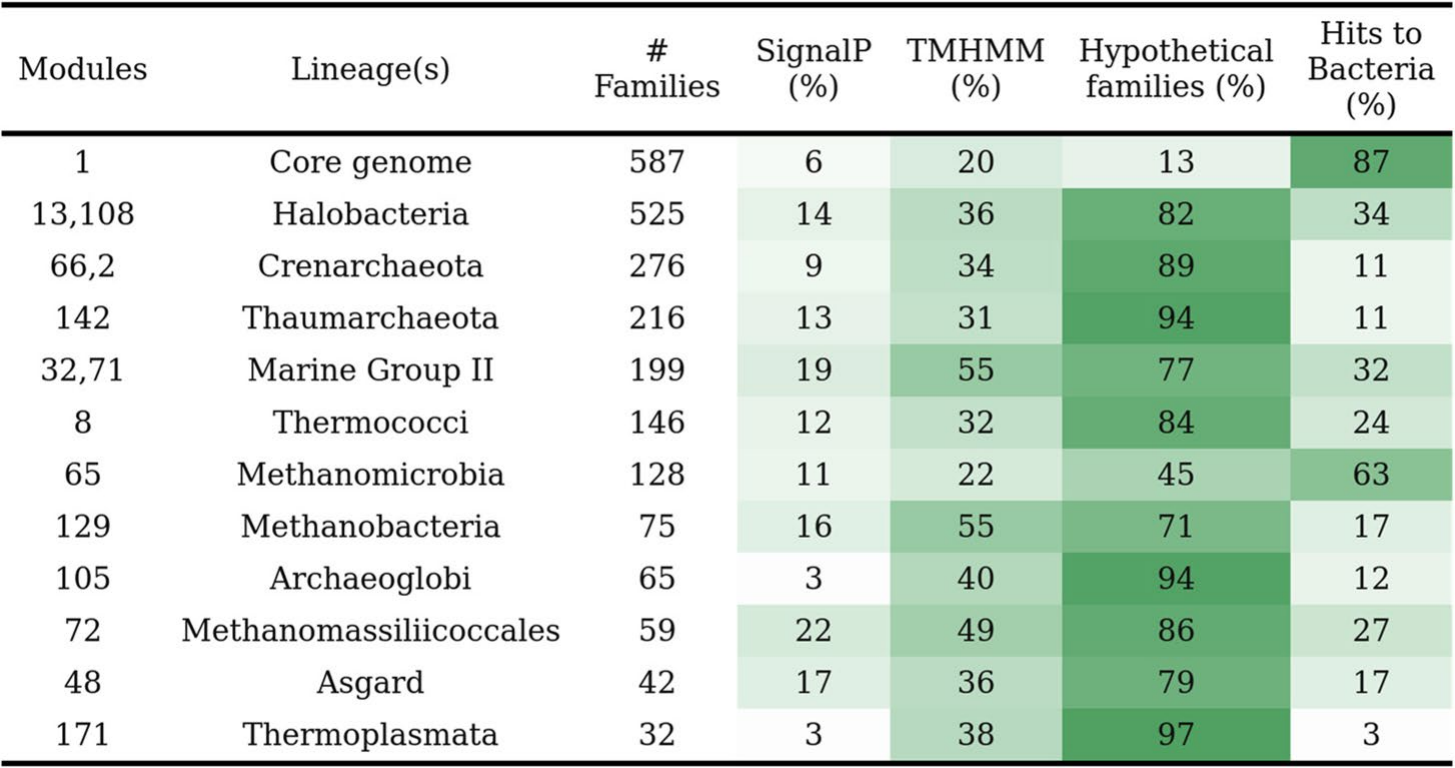
A list of the fourteen modules that are lineage specific but also well conserved within eleven major archaeal lineages. A family was counted as having a signal peptide if at least 25% of its protein sequences were predicted to have a signal peptide prediction according to the SignalP software [32]. A family was counted as having a transmembrane helix if more than half of its protein sequences were predicted to have a transmembrane helix according to the TMHMM software [33]. Families were considered hypothetical if they have neither PFAM (Domain of Unknown Function domains were excluded) nor KEGG annotations (see the supplementary dataset - Table S3 for the full list of hypothetical families). Finally, a family was considered to have bacterial homologs if the family matched with protein sequences of at least ten distinct bacterial genomes (see the “Methods” section). The core module 1 is included as a comparison

### Methanogens cluster together despite their phylogenetic diversity

We identified one module of 128 protein families, module 65 (Additional file 1: Table S3), that is common to essentially all methanogens, despite the fact that methanogens are not monophyletic [4]. This module contained *mcrA* (Fam05485), a key gene in methane production [34] all the other subunits (BCDG) of methyl–coenzyme M reductase (Mcr), five subunits of the methyl-tetrahydromethanopterin (methyl-H4MPT): coenzyme M methyltransferase (Mtr), five hypothetical conserved proteins in methanogens [35] and genes for transport of iron, magnesium, cobalt, and nickel and for synthesis of key cofactors that are required for growth of methanogens. Details are provided in Additional file 3.

Modules 72, 129, and 184 (for details, see Additional file 3) are enriched in subunits of the energy-converting hydrogenase A (group 4h) and B (group 4i) [36] and in enzymes for the utilization of methanol (fam04064 and fam05405), methylamine (fam02336 and fam03937), dimethylamine (fam03076 and fam05873), and trimethylamine (fam04092 and fam21299), which are substrates for methanogenesis [37].

Interestingly, we recovered mcr subunits in lineages that are not considered as canonical methanogenic lineages [38]. These include two genomes of Bathyarchaeota related to BA1 and BA2 (GCA_002509245.1 and GCA_001399805.1) [39], and one Archaeoglobi genome related to JdFR-42 (GCA_002010305) [40, 41]. These genomes have been described as having divergent MCR genes. It is reassuring that our method is sensitive enough to recover distant homology. Overall, the correspondence between the distribution of protein families linked to methanogenesis and methanogens supports the validity of our protein family delineation method (Additional file 2: Fig. S8).

### Functions specific to Poseidoniales

Modules 32 and 71, encompassing 199 families, were consistently associated with genomes of Poseidoniales, formerly Marine Group II (MGII) [42] (Additional file 1: Table S3), which are implicated in protein and saccharide degradation [43] (for details, see Additional file 3). These modules contain protein degrading enzymes (several different classes of peptidases and one oligotransporter) previously found in Poseidoniales [43] and two new Poseidoniales-specific families of well-conserved peptidases. As reported by Tully [43], peptidase S15 (PF02129; fam03321) and peptidase M60-like (PF13402; fam05454) have a narrow distribution within Poseidoniales, and were not assigned to ones of the 96 modules. Interestingly, we identified modules specific to Poseidoniales subgroup *Candidatus* Poseidonaceae (formerly subgroup MGIIa) (module 135, containing 99 families) and Poseidoniales subgroup *Candidatus* Thalassarchaeaceae (formerly subgroup MGIIb) (module 45, containing 39 families) with calcium-binding domains (Additional file 2: Fig. S9). These proteins may be involved in signaling and regulation of protein-protein interactions in the cell [44].

### Functions specific to Crenarchaeota

The Crenarchaeota comprises thermophilic organisms that are divided into three main classes, the Thermoproteales, the Sulfolobales, and the Desulfurococcales. Two distinct modules with distinct distributions were retrieved. Module 66 (61 families) is widespread in the three classes of Crenarchaeota whereas module 2 (215 families) is specific to the Sulfolobales class (Additional file 1: Table S3). Interestingly, the subunits of RNA polymerase [45], RpoG/Rpb8 (fam03177), are widespread in Crenarchaeota but Rpo13 (fam03159) seems restricted to the *Sulfolobales* class [45]. The Rpo13 protein family of Thermoproteales and Desulfurococcales may be highly divergent from the form described experimentally.

Comparison to PDB enabled annotation of three families with no PFAM and KEGG annotations as having functions related to the DNA replication machinery (Additional file 1: Table S4). We were interested to find that this ubiquitous function is performed by specific protein families in Crenarchaeota, possibly reflecting adaptation to their high-temperature habitats. One of these, PolB1-binding protein 2 (PBP2) (fam03141, PDB accession 5n35) [46], is a subunit of DNA polymerases B1 (PolB1) that are responsible for initial RNA primer extension with DNA, lagging and leading strand synthesis. The second is a single-stranded DNA-binding protein (DBP) ThermoDBP, which we also found to be conserved in Crenarchaeota and in Thermococci (fam03176, PDB accession 4psl) [47, 48]. Interestingly, however, the third is a Fe-S independent primase subunit PriX (fam03870, PDB accessions: 4wyh and 5of3) specific to Sulfolobales (Additional file 2: Fig. S10). PriX is essential for the growth of Sulfolobus cells [49, 50]. These observations point to fundamentally different transcription and replication mechanisms in the major groups within the Crenarchaeota.

Restricted to the Sulfolobales are also two multicopy thermostable acid protease thermopsin families [51] (fam01298 and fam01602 in module 2). Fam01298 is also found in two genomes of Thermoproteales (Additional file 2: Fig. S10). Extending a prior report that Crenarchaeota have anomalously large numbers of types I and III CRISPR-Cas systems [52], Crenarchaeota-specific module 66 contains four type I-A Cas families (one of which is the sulfolobales-specific CRISPR-associated protein csaX, fam07252) and four Cas families associated with type III systems (Additional file 2: Fig. S10).

### Functions specific to Thaumarchaeota

The phylum Thaumarcheaota mostly contains aerobic ammonia oxidizing archaea [4, 13]. Module 142, which contains 216 families, is specific to Thaumarchaeota. Although this module contains protein families for the three subunits of the ammonia monooxygenase, these three families are absent in genomes for two basal Thaumarcheota lineages, as expected based on prior analyses [4, 14] (Additional file 2: Fig. S11). This module also contains a highly conserved hypothetical family (fam08021), referred to as AmoX [53], that is known to co-occur with the amoABC genomic cluster (Additional file 1: Table S5). Importantly, essentially all other protein families in module 142 currently lack functional annotations (Additional file 3 and Additional file 1: Table S3).

### Functions specific to Thermococci

The Thermococci comprises sulfur-reducing hyperthermophilic archaea (Palaeococcus, Thermococcus, and Pyrococcus). Module 8 contains 146 families abundant in Thermococci and absent or sparsely distributed in other archaeal lineages (Additional file 1: Table S3). For example, 98% of the Thermococci genomes have a group 3b (NADP-reducing) [NiFe] hydrogenase whereas the group 3b hydrogenase is sparsely distributed in several other lineages such as in Asgard, Bathyarchaeota, Thermoplasmata, Methanomassiliicoccales, and Archaeoglobi. This hydrogenase, also known as sulfhydrogenase, is likely bidirectional [54]. Only the subunit beta of the sulfur reductase (fam04571) is present in module 8. Subunits alpha (fam00341), delta (fam00630), and gamma (fam00435) are present in the core module (module 1), probably because they are homologs of other hydrogenases. We also detected hydrogen gas-evolving membrane-bound hydrogenases (MBH) in every Thermococci genome (fam03754 in module 8) [55, 56]. The MBH transfers electrons from ferredoxin to reduce protons to form H_2_ gas [57]. The Na^+^-translocating unit of the MBH enables H_2_ gas evolution by MBH to establish a Na^+^ gradient for ATP synthesis near 100 °C in *Pyrococcus furiosus* [55]. As with the sulfhydrogenase, only the subunit I of the MBH is present in module 8, other subunits of MBH are present in core modules 1 and 23 probably because MBH-type respiratory complexes are evolutionarily and functionally related to the Mrp H+/Na+ antiporter system [55].

In the Thermococci-specific module 8, we detected the alpha and gamma subunits (represented by fam10869 and fam02435, respectively) of the Na^+^-pumping methylmalonyl-coenzyme A (CoA) decarboxylase that performs Na^+^ extrusion at the expense of the free energy of decarboxylation reactions [58, 59]. The beta and delta subunit, fam02317 and fam00273, are present in the core module 1, again probably because they are homologs of proteins that perform different functions.

Interestingly, three families from module 8 are encoded adjacent in the Thermococci genomes (fam15060, fam07594, and fam05926) (Additional file 1: Table S6). These are annotated as fungal lactamase (renamed prokaryotic 5-oxoprolinase A, pxpA) and homologs of allophanate hydrolase subunits (renamed pxpB and pxpC) and are likely to form together an 5-oxoprolinase complex [60]. While oxoproline is a major universal metabolite damage product and oxoproline disposal systems are common in all domains of life, the system encoded by these three families appears to be highly conserved in Thermococci.

We found the ribosomal protein L41e (fam02171) [61] in 83% of the genomes of Thermococci but sparsely distributed in DPANN, Poseidoniales, Hadesarchaea, Methanomassiliicoccales, and Pontarchaea or absent in other lineages. It has previously been noted that the distribution of L41e in Archaea is uncertain [62].

Using PDB, we established annotations for three families in Thermococci-specific module 8 that lacked PFAM or KEGG annotations (Additional file 1: Table S4). The first appears to be a small protein that inhibits the proliferating cell nuclear antigen by breaking the DNA clamp in *Thermococcus kodakarensis* (fam09868) [63]. The second is the S component of an energy-coupling factor (ECF) transporter (fam02033) likely responsible for vitamin uptake [64]. The protein sequences from the third (fam01133) show local similarities with the Valosin-containing protein-like ATPase (VAT) (fam00003) that in *Thermoplasma acidophilum* functions in concert with the 20S proteasome by unfolding substrates and passing them on for degradation [65]. Finally, three peptidases were detected in module 8 (fam01338, fam26972, and fam05052), thus may be specific to the Thermococci (Additional file 2: Fig. S12).

### Functions specific to Halobacteria

We found that 525 families comprise the Halobacteria-specific modules 13 and 108. Module 108 is composed almost completely of hypothetical proteins (Additional file 1: Table S3).

Module 13 contains the two subunits I (fam02395) and II (fam06634) of the high-affinity oxygen cytochrome *bd* oxidase (module 13) and was identified in half of the genomes. It also contains three families without KEGG and PFAM annotations, but close inspection using HMM-HMM comparison showed that they have distant homology with cytochrome-related proteins (Additional file 1: Table S4). The first, fam02696, has distant homology with the catalytic subunit I of heme-copper oxygen reductases (fam00581) and the genes often colocalize with heme-copper oxygen reductases-related genes such as type C (*cbb*_*3*_) subunit I or the nitric oxide reductase subunit B (fam00581) (Additional file 1: Table S7). The two other families are cytochrome c-associated proteins (fam01001, cytochrome c biogenesis factor and fam02143, Cytochrome C and Quinol oxidase polypeptide I). Consistent with the presence of oxygen respiration-related families, a catalase-peroxidase gene is present in 90% (fam02210) of the halobacteria genomes (Additional file 2: Fig. S13). Module 13 also includes proteins for synthesis of proteinaceous gas vacuoles (fam03834, fam03740, fam02854 and fam00889; identified in more than 45% of halobacterial genomes, Additional file 1: Table S3) that regulate buoyancy of cells in aqueous environments [66]. The module also includes bacterioruberin 2”, 3”-hydratase (fam00736, CruF; identified in 97% of the halobacteria genomes). Adjacent in the Halobacteria genomes are two families found in the core module 1 (fam00008 and fam00115) and annotated as digeranylgeranylglycerophospholipid reductase and UbiA prenyltransferases respectively (Additional file 1: Table S7). Closer inspection of these three co-encoded enzymes in *Haloarcula japonica* DSM 6131 (GCA_000336635.1) showed they are identical with the bifunctional lycopene elongase and 1,2-hydratase (LyeJ, fam00115) and the carotenoid 3,4-desaturase (CrtD, fam00008) and the bacterioruberin 2”, 3”-hydratase (CruF, fam00736) genes described in *Haloarcula japonica* JCM 7785^T^ [67]. Together, these three enzymes can generate C50 carotenoid bacterioruberin from lycopene in *Haloarcula japonica* [67]. Our results showed that C50 carotenoid bacterioruberin is highly conserved in Halobacteria (Additional file 2: Fig. S13).

### Functions specific to the six Asgard genomes

Module 48 contains 42 families that are specific and conserved in the six genomes of the superphylum Asgard (four genomes of Thorarchaeota and two genomes of Heimdallarchaeota). Of these, 33 lack both KEGG and PFAM functional predictions (Additional file 1: Table S3). The Asgard archaea, which affiliate with eukaryotes in the tree of life [7, 8], encode many proteins that they share with eukaryotes [68]. We detected four eukaryotic signature protein families (ESPs) in module 48 that were described in previous studies (Additional file 2: Fig. S14) [7, 8, 69].

**Fig. 3:**
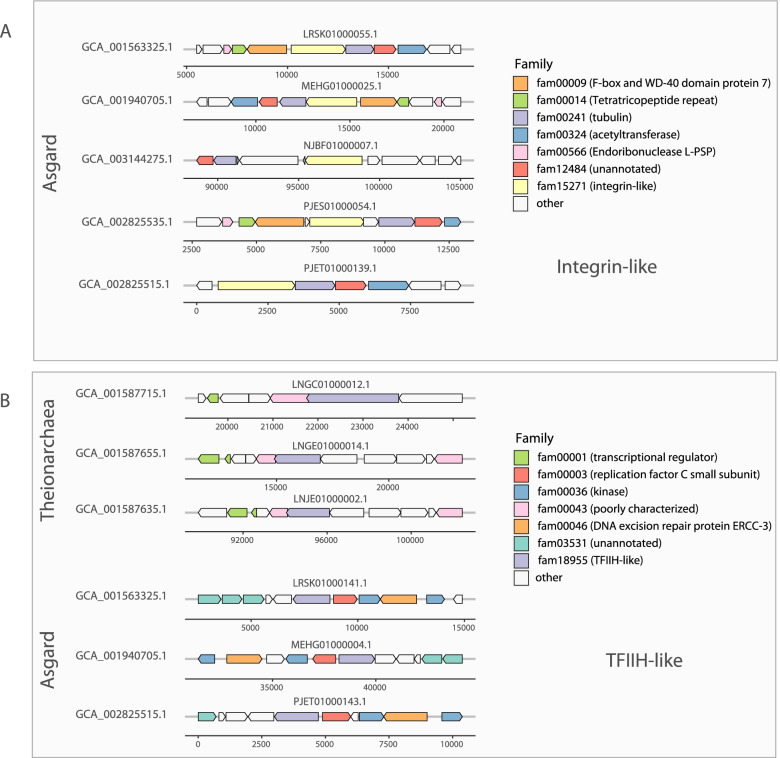
Schematic overview of integrin-like and TFIIH-like gene clusters identified in archaea. **A** Conserved gene clusters comprising archaeal integrin-like genes (fam15271 ) identified in five Asgard genomes. **B** Conserved gene clusters comprising archaeal TFIIH-like genes (fam18955) identified in three Theionarchaea and three Asgard genomes. A full gene synteny and genomic context of the genes neighboring the integrin-like (fam15271) and TFIIH-like (fam18955) genes is available in Additional file 1: Table S8

Interestingly, we found a family in module 48 (fam15271) that shows sequence similarity with the integrin beta 4. These proteins do not share sequence similarity with the integrin repeat-containing ESPs recently identified in Asgard genomes [70] and may represent a new ESP. The genes of fam15271 are always located next to tubulin/FtsZ genes (fam00241) in the five Asgard genomes (Fig. 3 and Additional file 1: Table S8). This is particularly interesting as recent studies have shed light on the crosstalk between integrin and the microtubule cytoskeleton [71]. Finally, one family in module 48 (fam18955) is annotated as the DNA excision repair protein ERCC-3 in three Asgard genomes and three Theionarchaea genomes. The genes neighboring the genes of fam18955 differ between the two lineages (Fig. 3) and the three Asgard sequences only share between 20 and 23% protein identity with the three Theionarchaea sequences. These differences may indicate two distinct functions for this family. Fam18955 shows distant homology with the protein RAD25 of *Saccharomyces cerevisiae*. RAD25 is a DNA helicase required for DNA repair and RNA polymerase II transcription in *S. cerevisiae* [72]. RAD25 is also one of the six subunits of the transcription factor IIH (TFIIH) in *S. cerevisiae* [73]. Consistent with the role of RAD25 in *S. cerevisiae*, the genes of family18955 are found next to replication factor C small subunit genes in the three Asgard genomes (Fig. 3 and Additional file 1: Table S8).

### Groups without lineage-specific metabolic signatures

The Archaeoglobi and Thermoplasmata lineages are unusual in that they have modules specific to them (modules 105 and 171 respectively), but no specific capacities were identified only in these groups based on functional predictions (Additional file 1: Table S3). These lineage-specific modules have the highest percentage of hypothetical families of any lineage-specific module (Table 1).

Bathyarchaeota is the only lineage having more than 10 genomes and that does not have a specific module of families that is widespread in the 19 Bathyarchaeota genomes (Additional file 1: Table S2). This is intriguing as Bathyarchaeota are widespread across terrestrial marine ecosystems and are known to thrive in diverse chemical environments [74].

## Hypothetical proteins distinguish the major archaeal groups

Even after augmenting functional predictions using PDB and EggNog databases, families with functional predictions represent a tiny proportion of the protein families that comprise the lineage-specific modules (Table 1 and Additional file 1: Table S3). In total, 1053 out of 1411 hypothetical families remain unannotated (Additional file 1: Table S4). A total of 358 hypothetical families have small domain matches but not enough information is available to predict functions. For example, many have domains with matches to zinc finger domains, but such domains occur in proteins with diverse functions (Additional file 1: Table S4). We found that the hypothetical proteins are shorter than proteins from the core families of module 1 (Additional file 2: Fig. S15) and are more likely to have a transmembrane helix prediction and a signal peptide prediction (Table 1).

Previous studies highlighted the presence of numerous families of proteins with roles in metabolism that are of bacterial origin but occur only in specific archaeal phyla [75, 76]. Consequently, we compared all archaeal protein families against a database of bacterial genomes sampled from across the bacterial tree of life to determine the extent to which proteins acquired from bacteria contribute to the archaeal group-specific modules (see the “Methods” section). From 3% (Thermoplasmata) to 34% (Halobacteria) of the protein families in modules that are archaeal group specific have homologs in ≥10 distinct bacterial genomes, with the exception of Methanomicrobia, where 63% of the protein families have bacterial homologs (Table 1). The hits are strikingly common to bacteria of the phylum Chloroflexi (Additional file 1: Table S3). We cannot offer an explanation, but it has been noted previously that a surprising number of Chloroflexi proteins have hits in Archaea [77]. Thus, for almost all archaeal groups, the majority of the protein families that form modules that separate them from other archaeal groups did not evolve in (or were not acquired from) bacteria. Furthermore, we conclude genes acquired from bacteria only account for a small fraction of the lineage-specific families although we cannot rule out the fact that high divergence between bacterial and archaeal protein sequences have erased sequence similarities.

## Discussion

We constructed a set of protein families for Domain Archaea, each of which generally corresponds with a set of homologous proteins with the same predicted function (in cases where functions could be assigned). Protein families with functional predictions that are specific to certain archaeal lineages (e.g., genes involved in methanogenesis or ammonia oxidation) well predict functional traits specific to these lineages. These observations indicate that the protein family construction method is robust. The generated set of 10,866 protein families is provided as an important community research resource. The patterns of presence/absence of protein families across genomes highlight sets of co-occurring proteins (modules), and groupings of genomes based on these modules mostly recapitulate archaeal phylogeny.

What is most striking from our analyses is the prominence of families of hypothetical proteins in the sets of highly prevalent lineage-specific proteins. An important consideration is whether (i) divergence of the sequence of these proteins from proteins with known function simply precluded functional annotation or (ii) whether they are novel proteins that serve well-known functions, or if (iii) they represent functions that are unique and evolved following the divergence of each lineage from other archaea. Our analyses were designed to avoid case (i) by relying on state-of-the-art HMM-based homology detection methods that appear to well-group proteins with shared functions (Additional file 2: Fig. S3). However, the fact that we could identify some probable functions using protein modeling suggests that (i) is correct in at least a subset of cases. For instance, PriX (fam03870) has structural homology with PriL but no sequence similarity was detected between PriX and any other protein in our analysis. Both proteins are distinct components of the primase complex in *Sulfolobus solfataricus* suggesting that PriX evolved from PriL by duplication followed by subfunctionalization [49, 50]. Lineage-specific hypothetical proteins that are actually homologs of known proteins but currently too divergent for functional assignment are interesting, as they may have been under pressure to evolve more rapidly than normal during lineage divergence. It is not possible to distinguish (ii) from (iii) with the data available. Both involve gene originations that do not rely on preexisting genes as a substrate for evolution. De novo gene originations have been under-studied in prokaryotes [78]. However, it is interesting to note that de novo protein candidates tend to be smaller and richer in predicted transmembrane domains than other proteins [78, 79] which is consistent with the features of lineage-specific proteins lacking predicted functions (Fig. S15 and Table 1). In general, the sets of relatively common archaeal proteins without functional assignments provide targets for future biochemical studies.

Overall, the prevalence of transmembrane helices and signal peptides in the hypothetical proteins in lineage-specific modules indicates that they are membrane associated or extracellular, thus possibly involved in cell-cell and cell-environment interactions (some may be transporters). Where the lineages are confined to specific environments (e.g., halophiles), lineage-specific protein families may have evolved to meet the requirements of that environment (case (i) or (iii)). It is important to note that some modules contain many protein families and probably represent combinations of new functions that, at the present time, cannot be resolved. Regardless of the explanation, or combination of explanations, for the presence of large numbers of lineage-specific proteins, the results indicate the importance of divergence or evolution of a specific subset of proteins during emergence of the major archaeal lineages.

Possibly also informative regarding archaeal evolution is the observation that, despite resolving a domain-wide core module (module 1), we detected only one case where a clearly defined module is conserved at the superphylum level. It is important to note that, with additional genomes, the two newly recognized Asgard phyla may be reclassified into a single phylum, eliminating this exception as recently proposed by Rinke and coworkers [80]. The apparent lack of protein family module support for superphyla may argue against the phyletic gradualism, in which one lineage gradually transforms into another, and favor the theory of cladogenesis, where a lineage splits into two distinct lineages [81]. We acknowledge that (i) modules containing fewer than 20 protein families (the cutoff used to define modules) or (ii) building orthologous instead of homologous families may highlight sets of families uniquely associated with superphyla and (iii) that some potentially important archaeal lineages such as in Asgard were not included in the current analysis due to lack of a sufficient number of high-quality genomes.

The observation that the patterns of presence/absence of shared protein families group together archaea that historically have been assigned to the same lineage and separate them from other lineages, in combination with innumerable prior publications on archaeal physiology and taxonomy [3–5], supports the value of the taxonomic classifications within Domain Archaea based on both phylogenetic tree of concatenated marker gene alignment and metabolic traits. Overall, the results suggest that early archaeal evolution rapidly generated the major lineages, the rise of which was linked to the acquisition of a set of proteins (recognized here as modules) that were largely retained during subsequent evolution of each lineage.

## Conclusions

Overall, we propose that hypothetical proteins were important in the origin of most of the major archaeal lineages and that the lack of blocks of protein families shared across superphyla is consistent with a burst-like origin of new lineages early in archaeal evolution.

## Methods

### Genome collection

A total of 569 unpublished genomes at the time we started the project [22] were added to the 2618 genomes of Archaea downloaded from the National Center for Biotechnology Information (NCBI) genome database in September 2018 (Additional file 1: Table S1).

One hundred thirty-two genomes were obtained from metagenomes of sediment samples. Sediment samples were collected from the Guaymas Basin (27° N 0.388, 111° W 24.560, Gulf of California, Mexico) during three cruises at a depth of approximately 2000 m below the water surface. Sediment cores were collected during two Alvin dives, 4486 and 4573 in 2008 and 2009. Sites referred to as “Megamat” (genomes starting with “Meg”) and “Aceto Balsamico” (genomes starting with “AB” in name), Core sections between 0 and 18 cm from 4486 and from 0 to 33 cm 4573 and were processed for these analyses. Intact sediment cores were subsampled under N_2_ gas and immediately frozen at −80 °C on board. The background of sampling sites was described previously [82]. Samples were processed for DNA isolation from using the MoBio PowerMax soil kit (Qiagen) following the manufacturer’s protocol. Illumina library preparation and sequencing were performed using Hiseq 4000 at Michigan State University. Paired-end reads were interleaved using interleav_fasta.py (https://github.com/jorvis/biocode/blob/master/fasta/interleave_fasta.py) and the interleaved sequences were trimmed using Sickle (https://github.com/najoshi/sickle) with the default settings. Metagenomic reads from each subsample were individually assembled using IDBA-UD with the following parameters: --pre_correction --mink 65 --maxk 115 --step 10 --seed_kmer 55 [83]. Metagenomic binning was performed on contigs with a minimum length of 2000 bp in individual assemblies using the binning tools MetaBAT [84] and CONCOCT [85], and resulting bins were combined with using DAS Tool [86]. CheckM lineage_wf (v1.0.5) [87] was used to estimate the percentage of completeness and contamination of bins. Genomes with more than 50% completeness and 10% contamination were manually optimized based on GC content, sequence depth, and coverage using mmgenome [88].

One hundred eighty-eight genomes were obtained from eight groundwater sites from Genasci Dairy Farm, located in Modesto, CA, USA [23]. Over 400 L of groundwater was filtered from monitoring wells on Genasci Dairy Farm over a period ranging from March 2017 to June 2018. DNA was extracted from all filters using Qiagen DNeasy PowerMax Soil kits and ~10 Gbp of 150-bp, paired-end Illumina reads was obtained for each filter. Assembly was performed using MEGAHIT [89] with default parameters, and the scaffolding function from assembler IDBA-UD was used to scaffold contigs. Scaffolds were binned on the basis of GC content, coverage, presence of ribosomal proteins, presence of single-copy genes, tetranucleotide frequency, and patterns of coverage across samples. Bins were obtained using manual binning on ggKbase [90], Maxbin2 [91], CONCOCT [85], Abawaca1, and Abawaca2 (https://github.com/CK7/abawaca), with DAS Tool [86] used to choose the best set of bins from all programs. All bins were manually checked to remove incorrectly assigned scaffolds using ggKbase.

Additionally, 168 genomes were obtained from an aquifer adjacent to the Colorado River near the town of Rifle, CO, USA, at the Rifle Integrated Field Research Challenge (IFRC) site [92]. Sediment samples were collected from the “RBG” field experiment carried out in 2007. Groundwater samples were collected from three different field experiments. All groundwater samples were collected from 5m below the ground surface by serial filtration onto 1.2, 0.2, and 0.1 μm filters (Supor disc filters; Pall Corporation, Port Washington, NY, USA). DNA was extracted from all frozen filters using the PowerSoil DNA Isolation kit (MoBio Laboratories Inc., Carlsbad, CA, USA) and 150-bp paired-end Illumina reads with a targeted insert size of 500 bp were obtained for each filter. Assemblies were performed using IDBA-UD [83] with the following parameters: --mink 40, --maxk 100, --step 20, --min_contig 500. All resulting scaffolds were clustered into genome bins using multiple algorithms. First, scaffolds were binned on the basis of % GC content, differential coverage abundance patterns across all samples using Abawaca1, and taxonomic affiliation. Scaffolds that did not associate with any cluster using this method were binned based on tetranucleotide frequency using Emergent Self-Organizing Maps (ESOM) [93]. All genomic bins were manually inspected within ggKbase.

Fifty genomes were obtained from the Crystal Geyser system in Utah, USA [94]. Microbial size filtration from Crystal Geyser fluids was performed using two different sampling systems. One system involved sequential filtration of aquifer fluids on 3.0-μm, 0.8-μm, 0.2-μm, and 0.1-μm filters (polyethersulfone, Pall 561 Corporation, NY, USA). The second system was designed to filter high volumes of water sequentially onto 2.5-μm, 0.65-μm, 0.2-μm, and 0.1-μm filters (ZTECG, Graver Technologies, Glasgow, USA). Metagenomic DNA was extracted from the filters using MoBio PowerMax soil kit. DNA was subjected to 150-bp paired-end illumina HiSeq sequencing at the Joint Genome Institute. Assembly of high-quality reads was performed using IDBA_UD with standard parameters and genes of assembled scaffolds (>1kb). Genome bins were obtained using different binning algorithms: semi-automated tetranucleotide-frequency-based emergent self-organizing maps (ESOMs), differential coverage ESOMs, Abawaca1, MetaBAT, and Maxbin2. Best genomes from each sample were selected using DAS Tool. All bins were manually checked to remove incorrectly assigned scaffolds using ggKbase.

Finally, forty-one genomes were obtained from the Uncultivated Bacteria and Archaea project [95] but were manually curated using ggKbase.

### Genome completeness assessment and de-replication

Genome completeness and contamination were estimated based on the presence of single-copy genes (SCGs). Genome completeness was estimated using 38 SCGs [96] (CCA-adding enzyme (COG1746), dimethyladenosine transferase (COG0030), diphthamide biosynthesis protein (COG1736), DNA-directed RNA polymerase (COG1095), DNA-directed RNA polymerase subunit N (COG1644), fibrillarin-like rRNA/tRNA 2′-O-methyltransferase (COG1889), glycyl-tRNA synthetase, KH type 1 domain protein (COG1094), methionyl-tRNA synthetase (COG0143), non-canonical purine NTP pyrophosphatase (COG0127), phenylalanyl-tRNA synthetase alpha subunit (COG0016), phenylalanyl-tRNA synthetase beta subunit (COG0072), pre-mRNA processing ribonucleoprotein (COG1498), prolyl-tRNA synthetase (COG0442), protein pelota homolog (COG1537), PUA domain containing protein (COG2016), ribosomal protein L10e (TIGR00279), ribosomal protein L13 (COG0102), ribosomal protein L18e (COG1727), ribosomal protein L21e (COG2139), ribosomal protein L3 (COG0087), ribosomal protein L7Ae/L8e (COG1358), ribosomal protein S13 (COG0099), ribosomal protein S15 (COG0184), ribosomal protein S19e (COG2238), ribosomal protein S2 (COG0052), ribosomal protein S28e (COG2053), ribosomal protein S3Ae (COG1890), ribosomal protein S6e (COG2125), ribosomal protein S7 (COG0049), ribosomal protein S9 (COG0103), ribosome maturation protein SDO1 homolog (COG1500), signal recognition particle 54 kDa protein (COG0541), transcription elongation factor Spt5 (TIGR00405), translation initiation factor 5A (COG0231), translation initiation factor IF-2 subunit gamma (COG5257), tRNA N6-adenosine threonylcarbamoyltransferase (COG0533), Valyl-tRNA synthetase (COG0525)). For non-DPANN archaea, genomes with more than 26 SCGs (>70% completeness) and less than 4 duplicated copies of the SCGs (<10% contamination) were considered as draft-quality genomes. Due to the reduced nature of DPANN genomes [9], DPANN genomes with more than 22 SCGs and less than 4 duplicated copies of the SCGs were considered as draft-quality genomes. Genomes were de-replicated using dRep [97] (version v2.0.5 with ANI > 95%). The most complete and less contaminated genome per cluster was used in downstream analyses.

### Concatenated 14 ribosomal proteins phylogeny

A maximum-likelihood tree was calculated based on the concatenation of 14 ribosomal proteins (L2, L3, L4, L5, L6, L14, L15, L18, L22, L24, S3, S8, S17, and S19). Homologous protein sequences were aligned using MAFFT (version 7.390) (--auto option) [98], and alignments refined to remove gapped regions using Trimal (version 1.4.22) (--gappyout option) [99]. The protein alignments were concatenated, with a final alignment of 1179 genomes and 2388 positions. The tree was inferred using RAxML [100] (version 8.2.10) (as implemented on the CIPRES web server [101]), under the LG plus gamma model of evolution, and with the number of bootstraps automatically determined via the MRE-based bootstopping criterion. A total of 108 bootstrap replicates were conducted, from which 100 were randomly sampled to determine support values.

### Protein clustering

Protein clustering into families was achieved using a two-step procedure. A first protein clustering was done using the fast and sensitive protein sequence searching software MMseqs2 (version 9f493f538d28b1412a2d124614e9d6ee27a55f45) [102]. An all-vs-all search was performed using *e*-value: 0.001, sensitivity: 7.5, and cover: 0.5. A sequence similarity network was built based on the pairwise similarities and the greedy set cover algorithm from MMseqs2 was performed to define protein subclusters. The resulting subclusters were defined as subfamilies. In order to test for distant homology, we grouped subfamilies into protein families using an HMM-HMM comparison procedure as follows: the proteins of each subfamily with at least two protein members were aligned using the result2msa parameter of mmseqs2, and, from the multiple sequence alignments, HMM profiles were built using the HHpred suite (version 3.0.3) [103]. The subfamilies were then compared to each other using hhblits [104] from the HHpred suite (with parameters -v 0 -p 50 -z 4 -Z 32000 -B 0 -b 0). For subfamilies with probability scores of ≥ 95% and coverage ≥ 0.50, a similarity score (probability × coverage) was used as weights of the input network in the final clustering using the Markov Clustering algorithm [105], with 2.0 as the inflation parameter. These clusters were defined as the protein families.

### Module definition and taxonomic assignment

Looking at the distribution of the protein families across the genomes, a clear modular organization emerged. Modules of families were defined using a cutoff of 0.95 on the dendrogram tree of the families. The dendrogram tree was obtained from a hierarchical clustering using the Jaccard distance that was calculated based on profiles of protein family presence/absence. The corresponding clusters define the modules.

A phyla distribution was assigned to each module using the method of [21]. Because modules contain genomes that carry only a few families of the modules, we designed a procedure to only identify genomes that carry most of the families of the modules. For each module, the median number of genomes per family (m) was calculated. The genomes were ranked by the number of families they carry. The m genomes that carry the most of families were retained; their phyla distribution defines the taxonomic assignment of the module.

### Functional annotation

Protein sequences were functionally annotated based on the accession of their best Hmmsearch match (version 3.1) (*E*-value cutoff 0.001) [106] against an HMM database constructed based on ortholog groups defined by the KEGG [24] (downloaded on June 10, 2015). The same hmmsearch procedure was used to annotate the protein sequences with the Unifam (Package_20170307) [28] and arCOG (ftp://ftp.ncbi.nih.gov/pub/wolf/COGs/arCOG/, accessed in February 2022) [27] databases. Domains were predicted using the same hmmsearch procedure against the Pfam database (version 31.0) [107]. The domain architecture of each protein sequence was predicted using the DAMA software (version 1.0) (default parameters) [108]. SIGNALP (version 5.0) (parameters: -format short -org arch) [32] was used to predict the putative cellular localization of the proteins. Prediction of transmembrane helices in proteins was performed using TMHMM (version 2.0) (default parameters) [33]. Protein sequences were also functionally annotated based on the accession of their best hmmsearch match (version 3.1) (*E*-value cutoff 1e−10) against the PDB database [25] (downloaded in February 2020).

### HMM-HMM predictions

Subfamilies were used to perform HMM-HMM annotation against arCogs of EggNog (version 5.0) [26] using hhblits [104] from the HHpred suite (with parameters -v 0 -p 50 -z 4 -Z 32000 -B 0 -b 0). Subfamilies were subsequently functionally annotated based on the EggNog accessions of their best probability score.

### Sequence similarity analysis

The 75,737 subfamilies from the 10,866 families were searched against a bacterial database of 2552 bacterial genomes [21] using hmmsearch (version 3.1) (*E*-value cutoff 0.001) [106]. Among them, 46,261 subfamilies, comprising 8300 families, have at least one hit against a bacterial genome.

## Supplementary Information

**Additional file 1: Table S1.** List of the 3197 genomes used in this study. For each genome (column A), its NCBI accession, GGKBASE link, number of scaffolds, genome size and number of CDS are displayed in columns B, C, D, E and F respectively. Genome source is in column G, dRep cluster in column H. Genome completeness and the contamination based on single copy genes are displayed in columns I and J respectively. Column K informs about the concatenated ribosomal proteins. The 1,179 representative genomes are indicated in column L. The phylum and superphylum (DPANN and non-DPANN) taxonomy of the representative genomes are provided in columns M and N. Taxonomy based on the different databases we pulled out the genomes is shown in column O. **Table S2.** Taxonomy distribution of the 113 modules. Module name is indicated in column A whereas the number of families is indicated in column B. Suggested taxonomic distribution is indicated in column C. Column D details the genomes used to define the taxonomic distribution (phylum, number of genomes). **Table S3.** Annotation of the 10,866 families. Column A: module number. Column B: family accession. Column C: number of proteins in the family. Column D: median length of the proteins. Column E: ratio of proteins predicted to contain a signal peptide. Column F: median number of predicted transmembrane helix per protein. Column G: domain architecture reported by Pfam. Columns H, I, J, K, L: KEGG annotations. Column M: Cazy annotation. Column N: arCOG annotation. Column O: Unifam annotation. Columns Q to AF indicate the ratio of genomes having the given family in the given archaeal phylum. Columns AG to CN indicate the ratio of genomes having the given family in the given bacterial phylum. **Table S4.** Annotation of the subfamilies (column C) based on Hmmsearch against the PDB database (columns D and E) and based on HMM-HMM prediction against the arCogs of the EggNOGs database (columns F, G, H and I). **Table S5.** Genes neighboring the four genes encoding the subunits of the ammonia monooxygenase. The four genes downstream and upstream of each amoA, amoB, amoC and amoX genes (column H) were identified and annotated using the protein clustering (column E), the PFAM (column G) and the KEGG databases (column F). **Table S6.** Genes neighboring the three genes encoding the subunits of the 5-oxoprolinase complex. The three genes downstream and upstream of each pxpA, pxpB and pxpC genes (column H) were identified and annotated using the protein clustering (column E), the PFAM (column G) and the KEGG databases (column F). **Table S7.** Genes neighboring the three genes encoding the enzymes of the pathway of the C50 carotenoid bacterioruberin and the gene encoding a distant homolog of the catalytic subunit I of heme-copper oxygen reductase (fam02696). The four genes downstream and upstream of each LyeJ, CruF, CrtD and fam02696 genes (column H) were identified and annotated using the protein clustering (column E), the PFAM (column G) and the KEGG databases (column F). **Table S8.** Genes neighboring the two genes encoding the integrin beta 4 and the TFIIH. The five genes downstream and upstream of each integrin and the TFIIH genes (column H) were identified and annotated using the protein clustering (column E), the PFAM (column G) and the KEGG databases (column F).

**Additional file 2: Figure S1.** Taxonomic assessment and distribution of the 1,179 representative genomes. Maximum-likelihood phylogeny based on a 14-ribosomal-protein concatenated alignment (2,388 positions) using the LG plus gamma model of evolution. Scale bar indicates the average substitutions per site. **Figure S2.** The protein clustering pipeline used in the study. MAGs: metagenome-assembled Genomes. **Figure S3.** Quality assessment of the protein clustering. A. Consistency between the KEGG annotations and the protein families. For each of the 6482 annotations, we reported the family which contains the highest percentage of protein members annotated with that KEGG annotation. Each dot represents a KEGG annotation, the y-axis represents the highest percentage. C. Contamination of the protein families. For each family with proteins having KEGG annotations, we computed the percentage of the proteins that have KEGG annotations different than the most abundant one, this percentage defined the annotation admixture (y-axis). Each dot represents a protein family. **Figure S4.** Comparison between the protein clustering performed in this study and the Unifam and the arCOG databases. The Venn diagram shows the number of ORFs within the 1,179 genomes that were clustered into families defined in this study (purple) and that have hits with arCOG (green) and Unifam (yellow) HMMs. **Figure S5.** Correlation plot of 4 trees obtained from 3 different hierarchical clustering methods (complete linkage, average linkage and single linkage). Maximum-likelihood tree based on RAxML is also shown (“Phylogenetic tree”). Correlations are based on cophenetic distance matrices between pairs of trees. Positive correlations are displayed in blue and negative correlations in red color. Color intensity is proportional to the correlation coefficient. **Figure S6.** The distribution of 10,866 widely distributed protein families (columns) in 1,179 representative genomes (rows) from Archaea. The families of the 19 modules discussed in the study were colored. Data are clustered based on the presence (black) and absence (white) profiles (Jaccard distance, complete linkage). **Figure S7.** Number of genomes per family in 14 selected modules. X-axis represents the families and y-axis the number of genomes. For each family, the number of genomes of Methanomicrobia, Methanobacteria, Halobacteria, Crenarchaeota, Poseidoniales (Marine group II), Thermococci, Archaeoglobi, Thaumarchaeota, Thermoplasmata and Methanomassiliicoccales are shown by a colored dot. **Figure S8.** Presence and absence of 37 families of modules 65, 72, 129 and 184 in genomes of methanogen archaea. Scale bar indicates the average substitutions per site. **Figure S9.** Presence and absence of 11 families of modules 32, 45, 71, 135 in the genomes of Poseidoniales. Scale bar indicates the average substitutions per site. **Figure S10.** Presence and absence of 15 families of modules 2 and 66 in genomes of Crenarchaeota. Scale bar indicates the average substitutions per site. **Figure S11.** Presence and absence of 4 families of module 142 in genomes of Thaumarchaeota. Scale bar indicates the average substitutions per site. **Figure S12.** Presence and absence of 14 families of module 8 in genomes of Thermococci. Scale bar indicates the average substitutions per site. **Figure S13.** Presence and absence of 11 families of modules 13 and 108 in genomes of Halobacteria. Scale bar indicates the average substitutions per site. **Figure S14.** Presence and absence of 6 families of module 48 in genomes of Asgard. Scale bar indicates the average substitutions per site. **Figure S15.** The length distribution of hypothetical proteins (in amino acid).

**Additional file 3.**

## Data Availability

The datasets presented in this study can be found in online repositories. The names of the repository/repositories and accession number(s) of the DPANN MAGs can be found below: NCBI BioProject, PRJNA288027 [109] and PRJNA692327 [110]. Individual accessions are provided in Additional file 1: Table S1. Detailed information of the genomes is provided in Additional file 1: Table S1. Detailed annotations of the families are provided in Additional file 1: Table S3 accompanying this paper. Raw data files (phylogenetic tree and fasta sequences of the families) are made available via figshare under the following link: 10.6084/m9.figshare.12676421 [111].

## Abbreviations

TACK: Thaumarchaeota, Aigarchaeota, Crenarchaeota, and Korarchaeota
DPANN: Diapherotrites, Parvarchaeota, Aenigmarchaeota, Nanoarchaeota, Nanohaloarchaea
MAGs: Metagenome-assembled genomes
ANI: Average nucleotide identity
KEGG: Kyoto Encyclopedia of Genes and Genomes
HMM: Hidden Markov model
arCOG: Archaeal Clusters of Orthologous Genes
CRISPR: Clustered regularly interspaced short palindromic repeats
MGII: Marine Group II
PDB: Protein Data bank
ESPs: Eukaryotic signature proteins
GTDB: Genome Taxonomy DataBase
NCBI: National Center for Biotechnology Information
ESOM: Emergent Self-Organizing Maps
SCGs: Single-copy genes
