# Supplementary material for: Conserved and lineage-specific hypothetical proteins may have played a central role in the rise and diversification of major archaeal groups
Source: BMC Biol. 2022 Jul 5;20:154. doi: 10.1186/s12915-022-01348-6 (PMC9258230; doi:10.1186/s12915-022-01348-6)
Supplement: Supplementary file 2 — Additional file 2: Figure S1. Taxonomic assessment and distribution of the 1,179 representative genomes. Maximum-likelihood phylogeny based on a 14-ribosomal-protein concatenated alignment (2,388 positions) using the LG plus gamma model of evolution. Scale bar indicates the average substitutions per site. Figure S2. The protein clustering pipeline used in the study. MAGs: metagenome-assembled Genomes. Figure S3. Quality assessment of the protein clustering. A. Consistency between the KEGG annotations and the protein families. For each of the 6482 annotations, we reported the family which contains the highest percentage of protein members annotated with that KEGG annotation. Each dot represents a KEGG annotation, the y-axis represents the highest percentage. C. Contamination of the protein families. For each family with proteins having KEGG annotations, we computed the percentage of the proteins that have KEGG annotations different than the most abundant one, this percentage defined the annotation admixture (y-axis). Each dot represents a protein family. Figure S4. Comparison between the protein clustering performed in this study and the Unifam and the arCOG databases. The Venn diagram shows the number of ORFs within the 1,179 genomes that were clustered into families defined in this study (purple) and that have hits with arCOG (green) and Unifam (yellow) HMMs. Figure S5. Correlation plot of 4 trees obtained from 3 different hierarchical clustering methods (complete linkage, average linkage and single linkage). Maximum-likelihood tree based on RAxML is also shown (“Phylogenetic tree”). Correlations are based on cophenetic distance matrices between pairs of trees. Positive correlations are displayed in blue and negative correlations in red color. Color intensity is proportional to the correlation coefficient. Figure S6. The distribution of 10,866 widely distributed protein families (columns) in 1,179 representative genomes (rows) from Archaea. The families of the 19 m [file 12915_2022_1348_MOESM2_ESM.pdf]

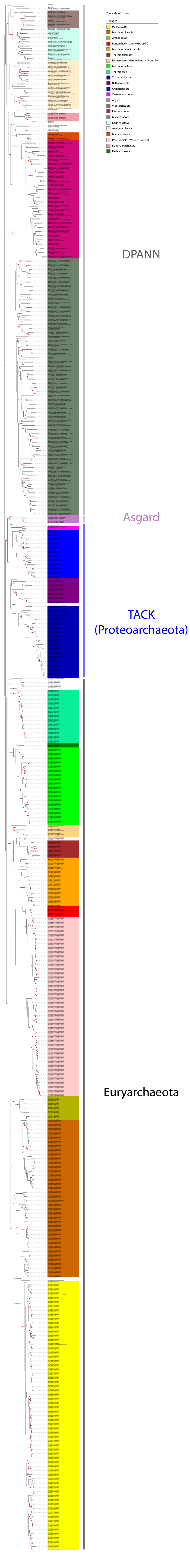

Tree scale: 0.1
Lineages
Halobacteria
Methanomicrobia
Archaeoglobi
Pontarchaea (Marine Group III)
Methanomassiliicoccales
Thermoplasmata
Izemarchaea (Marine Benthic Group D)
Methanobacteria
Thermococci
Thaumarcheota
Bathyarcheota
Crenarchaeota
Vestraetearcheota
Asgard
Woesarcheaota
Paecearcheota
Micrarchaeota
Diapherotrites
Aenigmarcheota
Mamarchaeota
Poseidoniales (Marine Group II)
Nanohaloarchaeota
Hadearchaeota
DPANN
Asgard
TACK
(Proteoarchaeota)
Euryarchaeota

Supplementary Figure S1. Taxonomic assessment and distribution of the 1,179 representative genomes. Maximum-likelihood phylogeny based on a 14-ribosomal-protein concatenated alignment (2,388 positions) using the LG plus gamma model of evolution. Scale bar indicates the average substitutions per site.

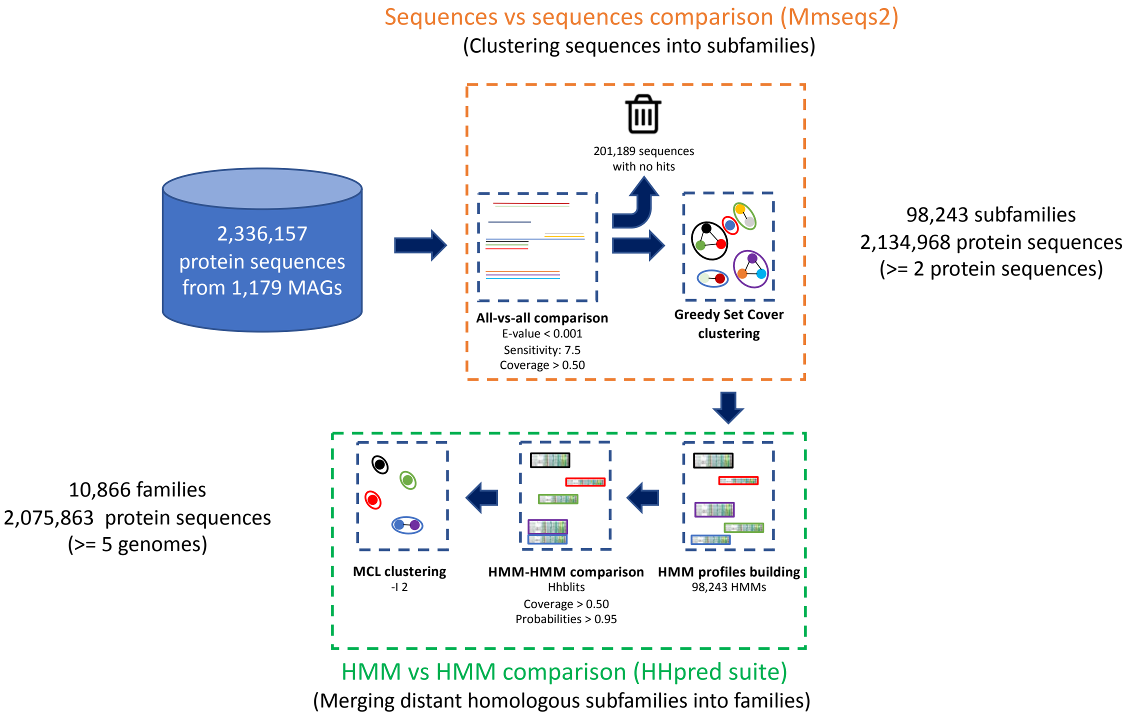

Figure S2. The protein clustering pipeline used in the study. MAGs: metagenome-assembled genomes.

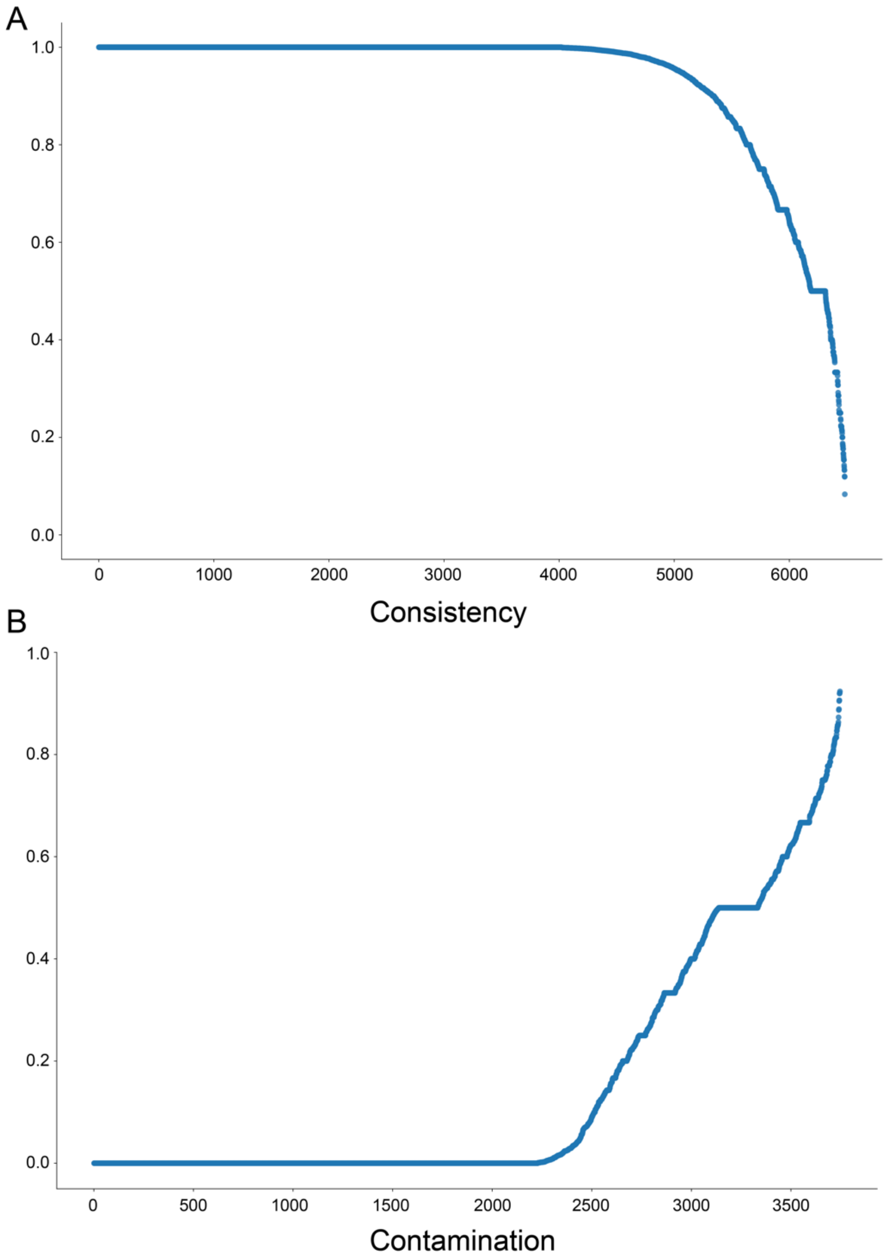

Figure S3. Quality assessment of the protein clustering. A. Consistency between the KEGG annotations and the protein families. For each of the 6482 annotations, we reported the family which contains the highest percentage of protein members annotated with that KEGG annotation. Each dot represents a KEGG annotation, the y-axis represents the highest percentage. C. Contamination of the protein families. For each family with proteins having KEGG annotations, we computed the percentage of the proteins that have KEGG annotations different than the most abundant one, this percentage defined the annotation admixture (y-axis). Each dot represents a protein family.

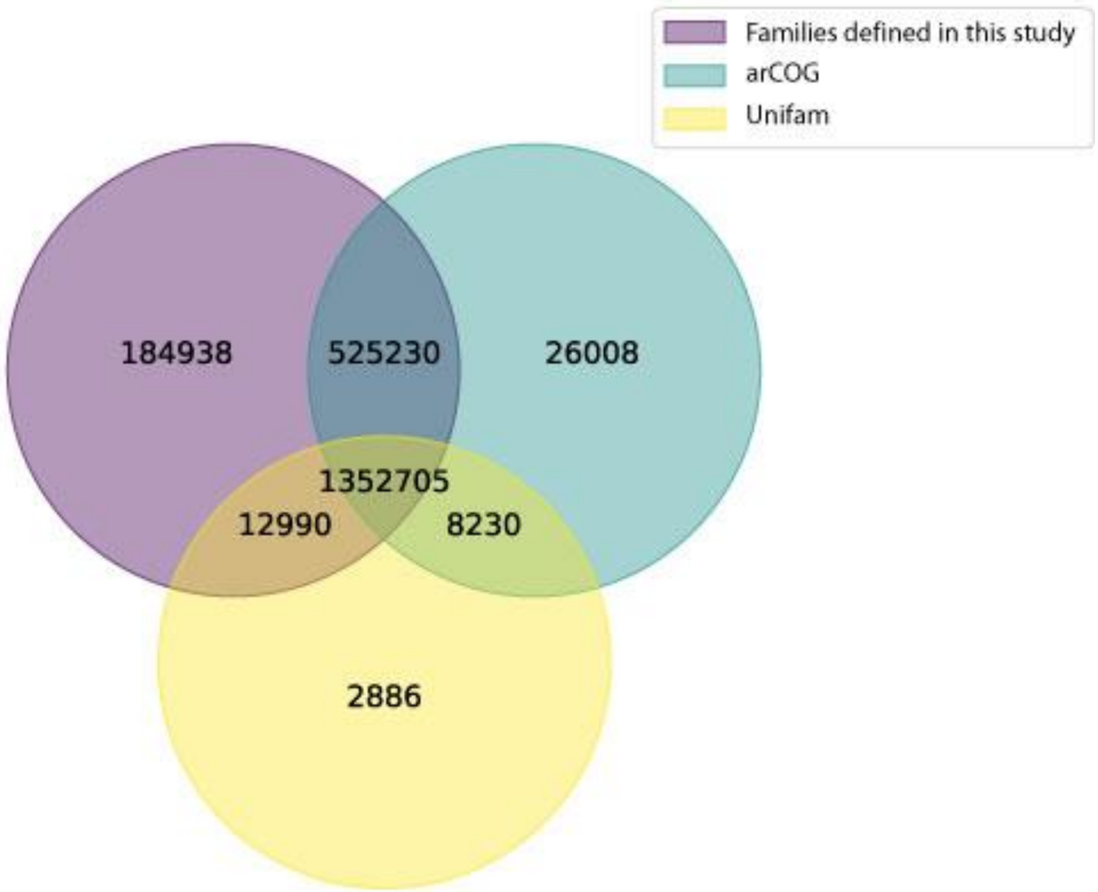

Figure S4. Comparison between the protein clustering performed in this study and the Unifam and the arCOG databases. The Venn diagram shows the number of ORFs within the 1,179 genomes that were clustered into families defined in this study (purple) and that have hits with arCOG (green) and Unifam (yellow) HMMs.

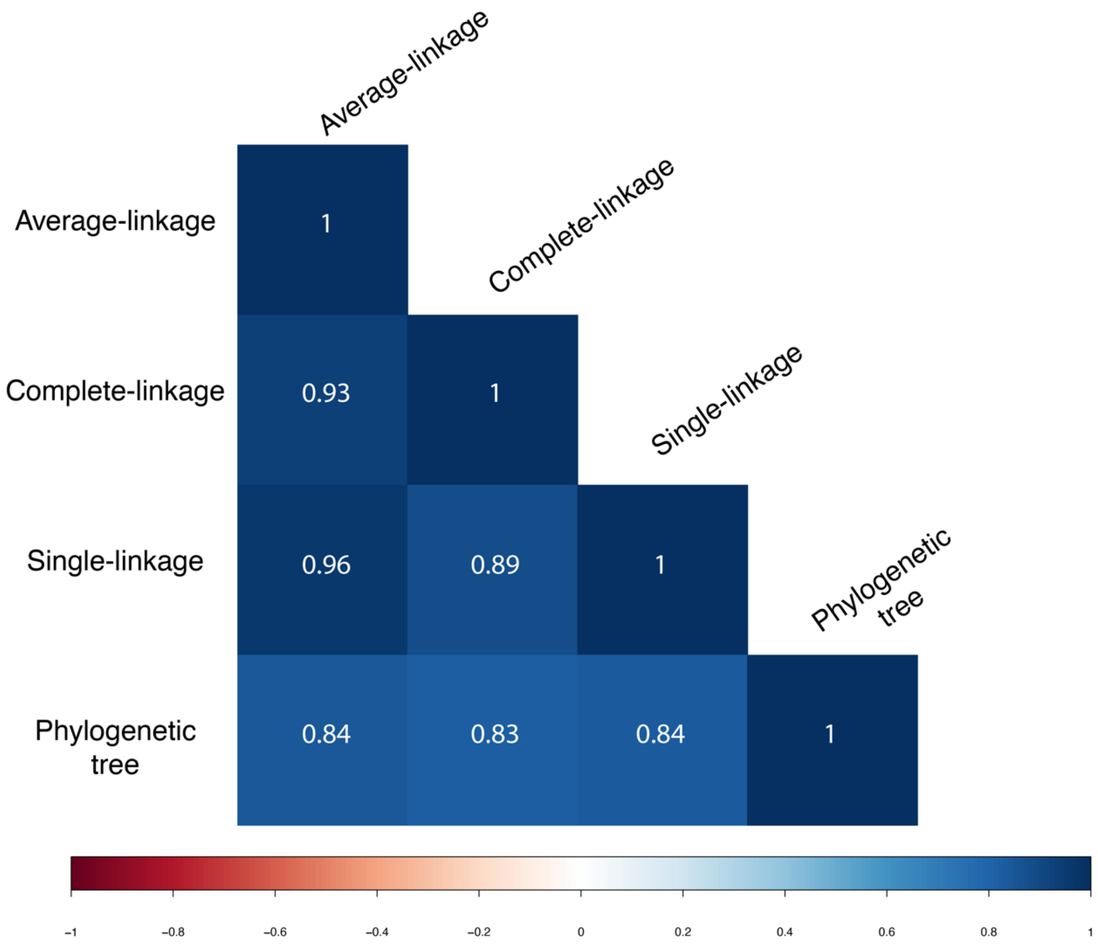

Supplementary Figure S5. Correlation plot of 4 trees obtained from 3 different hierarchical clustering methods (complete linkage, average linkage and single linkage). Maximum-likelihood tree based on RAxML is also shown ("Phylogenetic tree"). Correlations are based on cophenetic distance matrices between pairs of trees. Positive correlations are displayed in blue and negative correlations in red color. Color intensity is proportional to the correlation coefficient.

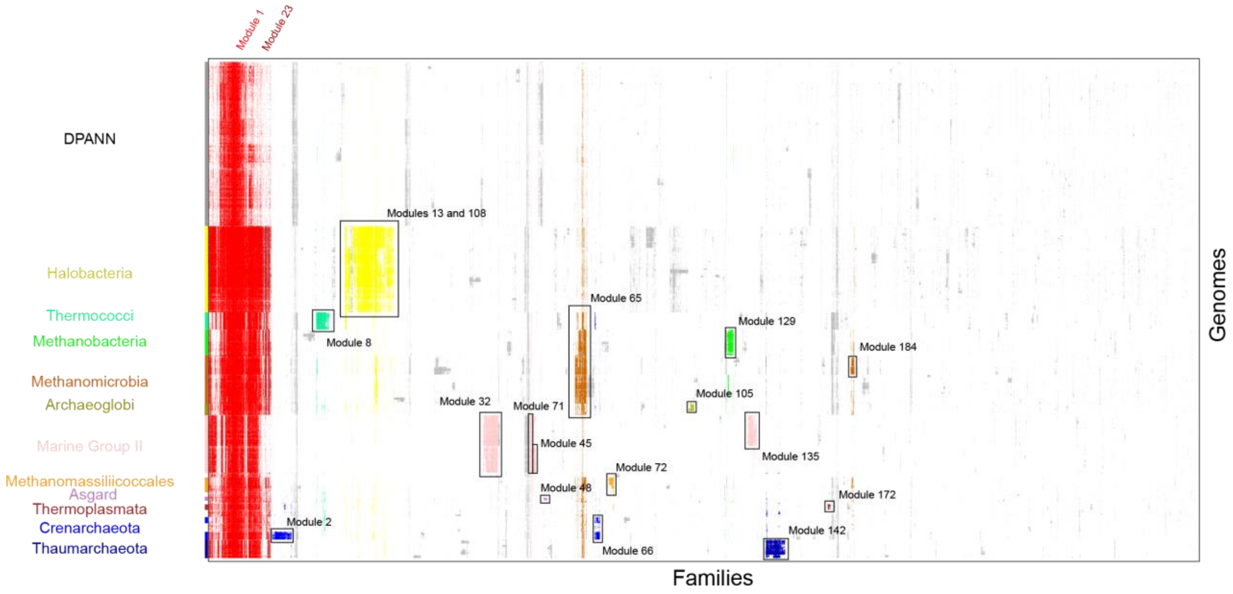

Supplementary Figure S6. The distribution of 10,866 widely distributed protein families (columns) in 1,179 representative genomes (rows) from Archaea. The families of the 19 modules discussed in the study were colored. Data are clustered based on the presence (black) and absence (white) profiles (Jaccard distance, complete linkage).

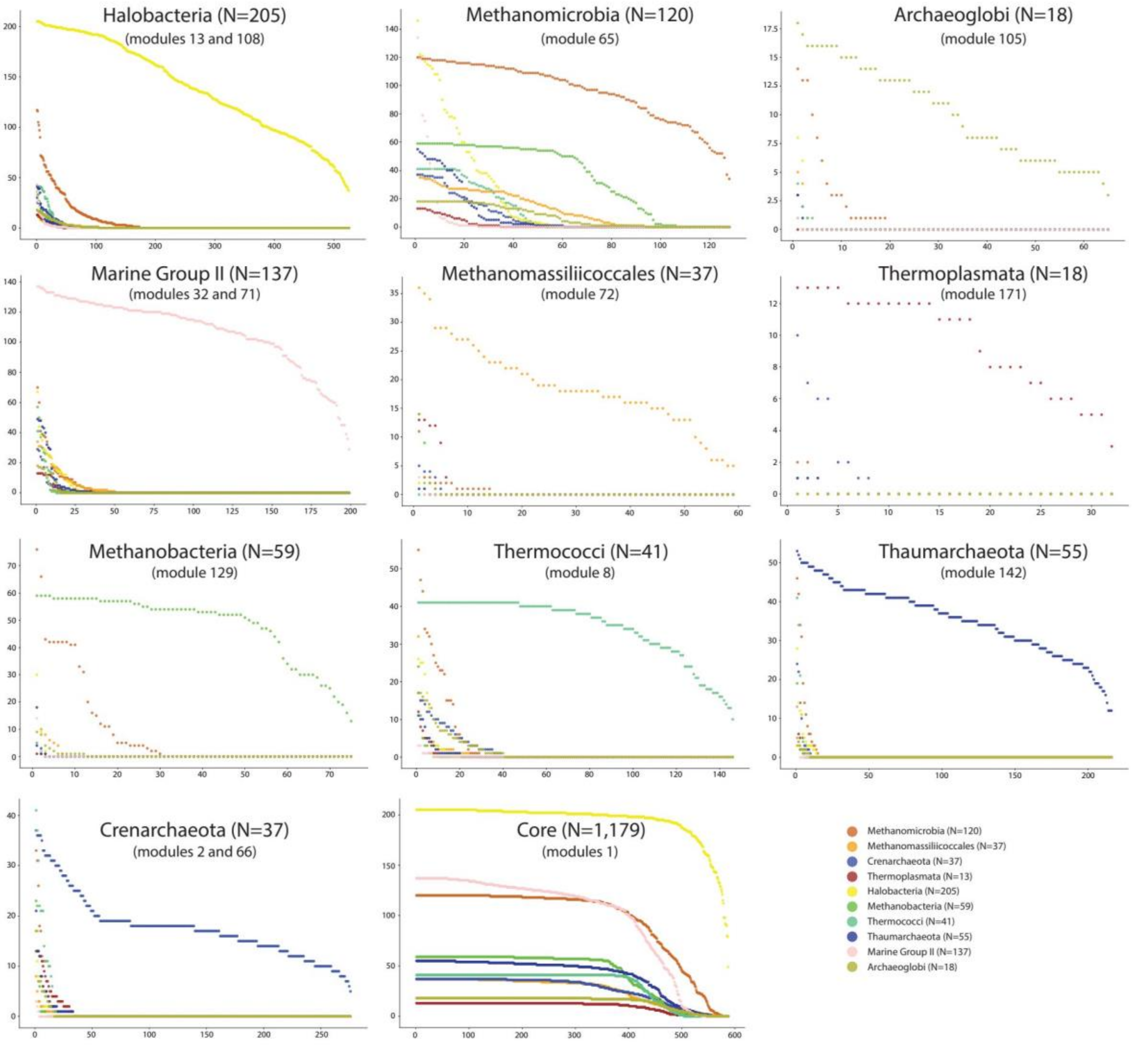

Supplementary Figure S7. Number of genomes per family in 14 selected modules. X-axis represents the families and y-axis the number of genomes. For each family, the number of genomes of Methanomicrobia, Methanobacteria, Halobacteria, Crenarchaeota, Poseidoniales (Marine group II), Thermococci, Archaeoglobi, Thaumarchaeota, Thermoplasmata and Methanomassiliicoccales is shown by a colored dot.

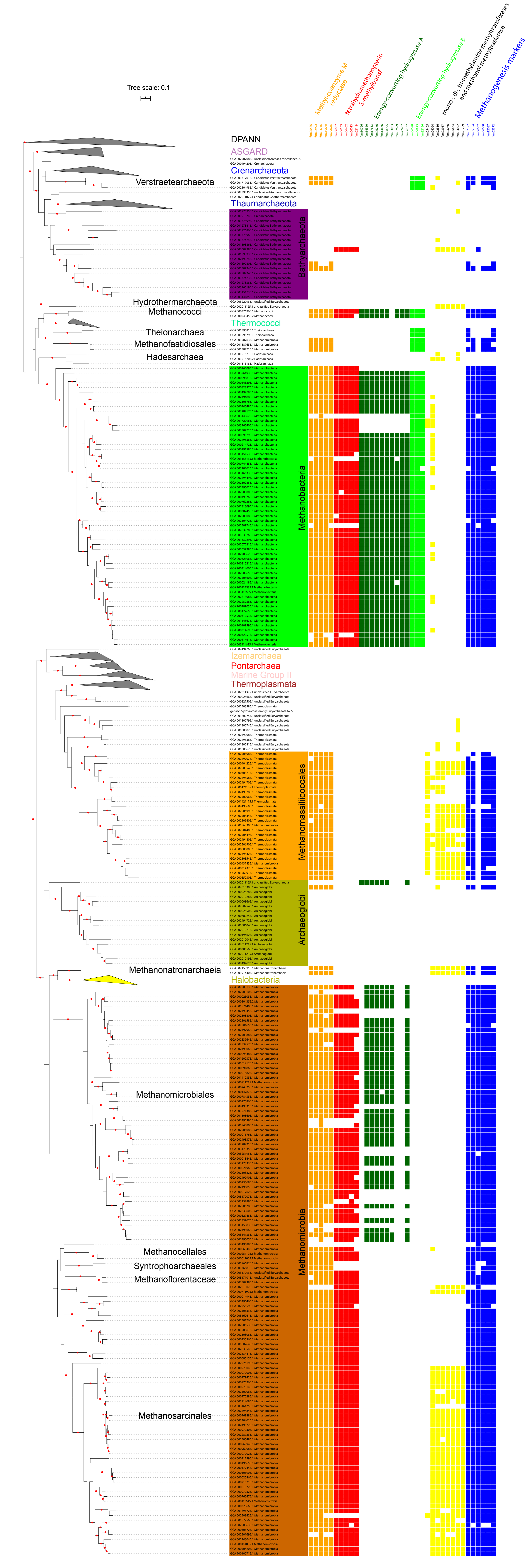
Tree scale: 0.1
Methyl-coenzyme M reductase
tetrahydromethanopterin S-methyltransf
Energy-converting hydrogenase A
Energy-converting hydrogenase B
mono-, di-, tri-methylamine methyltransferases and methanol methyltransferase
Methanogenesis markers
DPANN
ASGARD
Crenarchaeota
Verstraetearchaeota
Thaumarchaeota
Bathyarchaeota
Hydrothermarchaeota
Methanococci
Thermococci
Theionarchaea
Methanofastidiosales
Hadesarchaea
Methanobacteria
Izemarchaea
Pontarchaea
Marine Group II
Thermoplasmata
Methanomassiliicoccales
Archaeoglobi
Methanonatronarchaeia
Halobacteria
Methanomicrobiales
Methanocellales
Syntrophoarchaeales
Methanoflorentaceae
Methanosarcinales
Methanomicrobia

Supplementary Figure S8. Presence and absence of 37 families of modules 65, 72, 129 and 184 in genomes of methanogen archaea. Scale bar indicates the average substitutions per site.

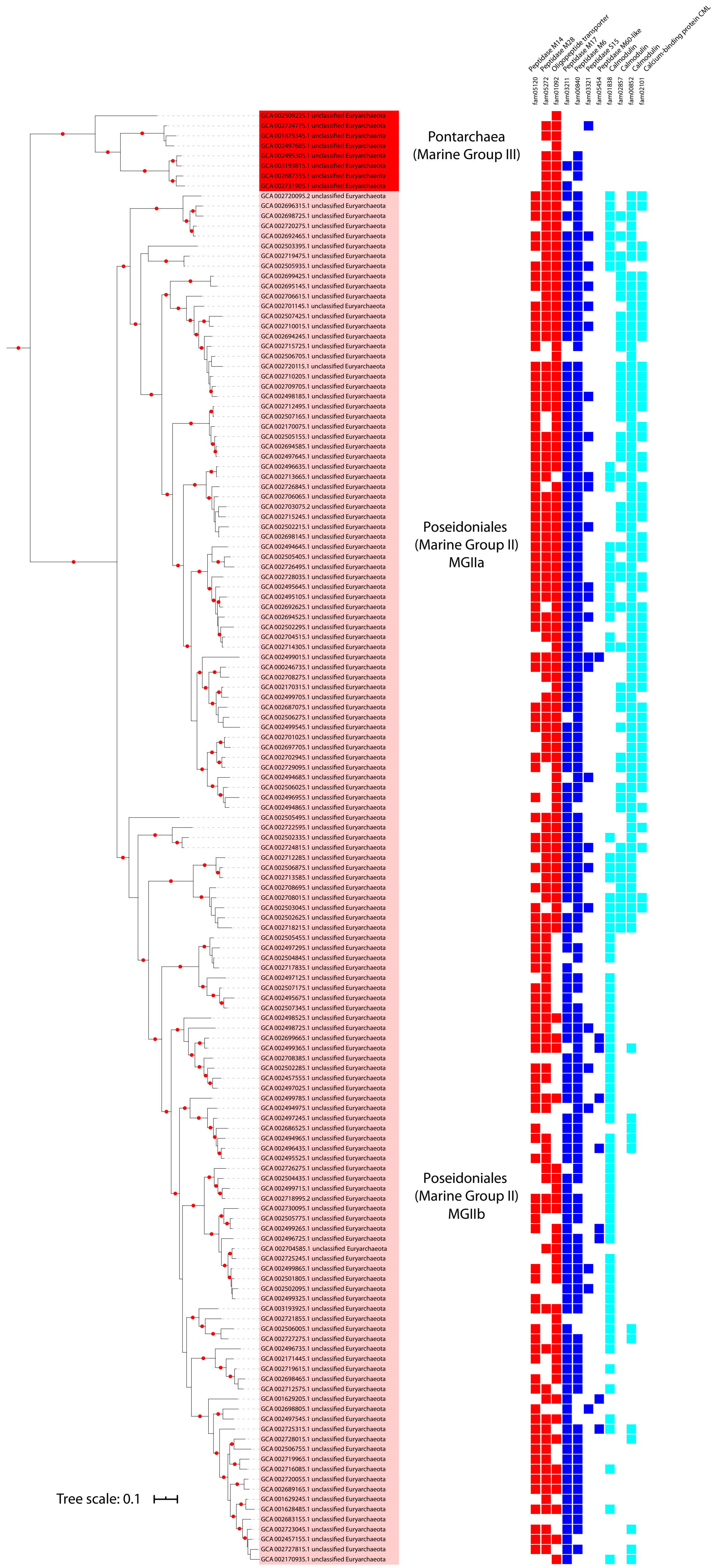
Peptidase M14
Peptidase M28
Oligopeptide transporter
Peptidase M17
Peptidase M6
Peptidase S15
Peptidase M60-like
Calmodulin
Calmodulin
Calmodulin
Calcium-binding protein CML
fam05120
fam05272
fam01092
fam03211
fam00840
fam03321
fam05454
fam01838
fam02857
fam00852
fam02101
Pontarchaea
(Marine Group III)
Poseidoniales
(Marine Group II)
MGIIa
Poseidoniales
(Marine Group II)
MGIIb
Tree scale: 0.1

Supplementary Figure S9. Presence and absence of 11 families of modules 32, 45, 71, 135 in the genomes of Poseidoniales. Scale bar indicates the average substitutions per site.

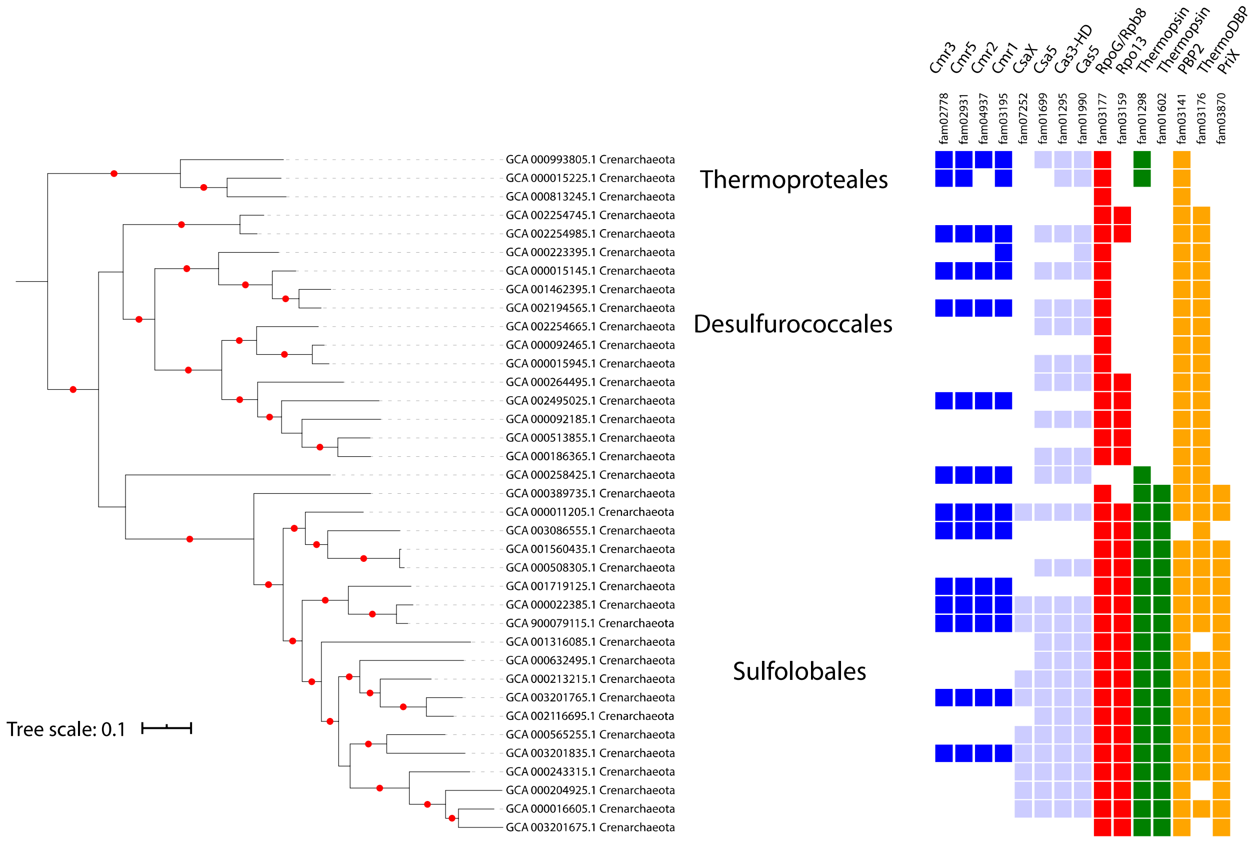

Supplementary Figure S10. Presence and absence of 15 families of modules 2 and 66 in genomes of Crenarchaeota. Scale bar indicates the average substitutions per site.

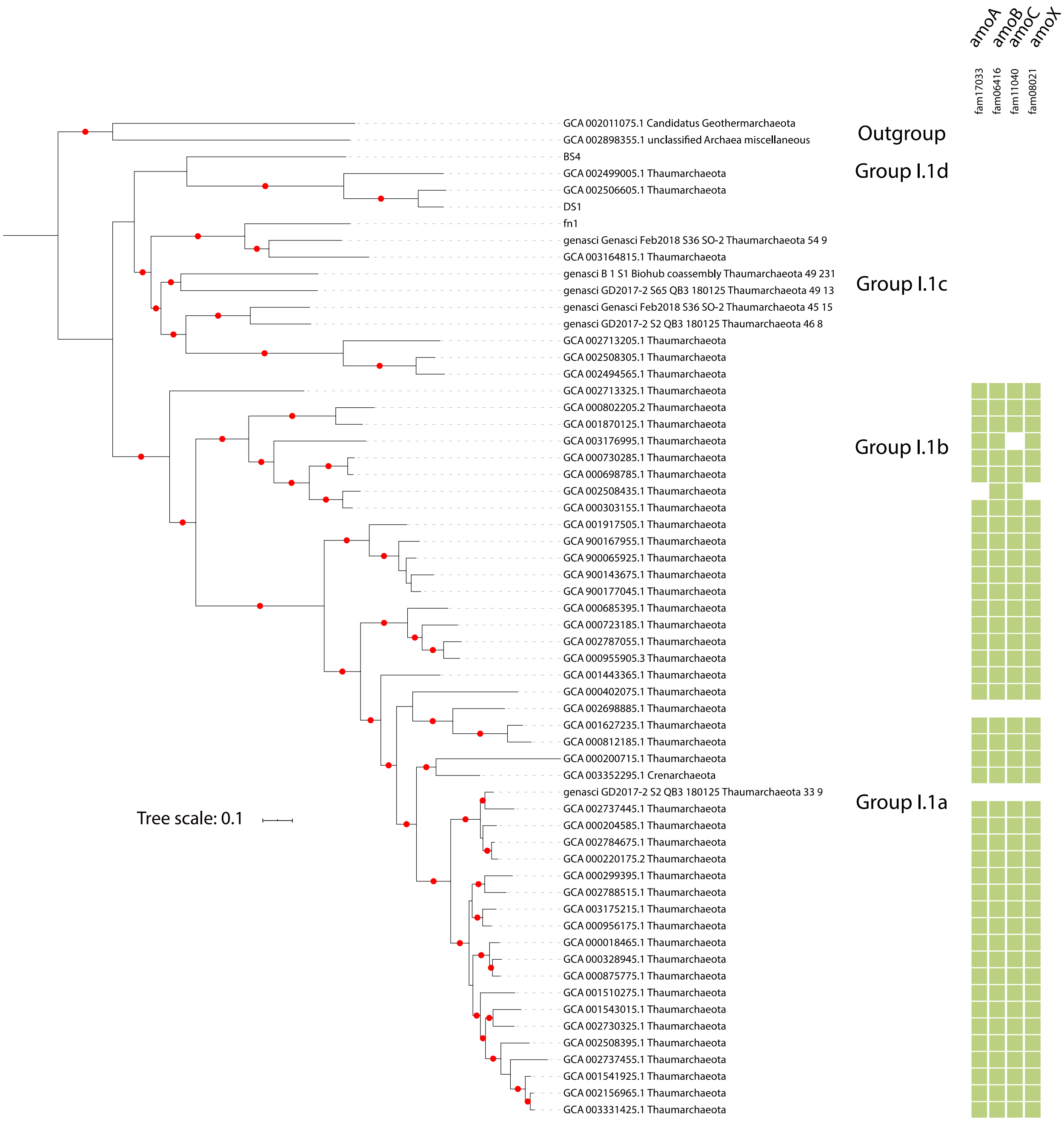

Supplementary Figure S11. Presence and absence of 4 families of module 142 in genomes of Thaumarchaeota. Scale bar indicates the average substitutions per site.

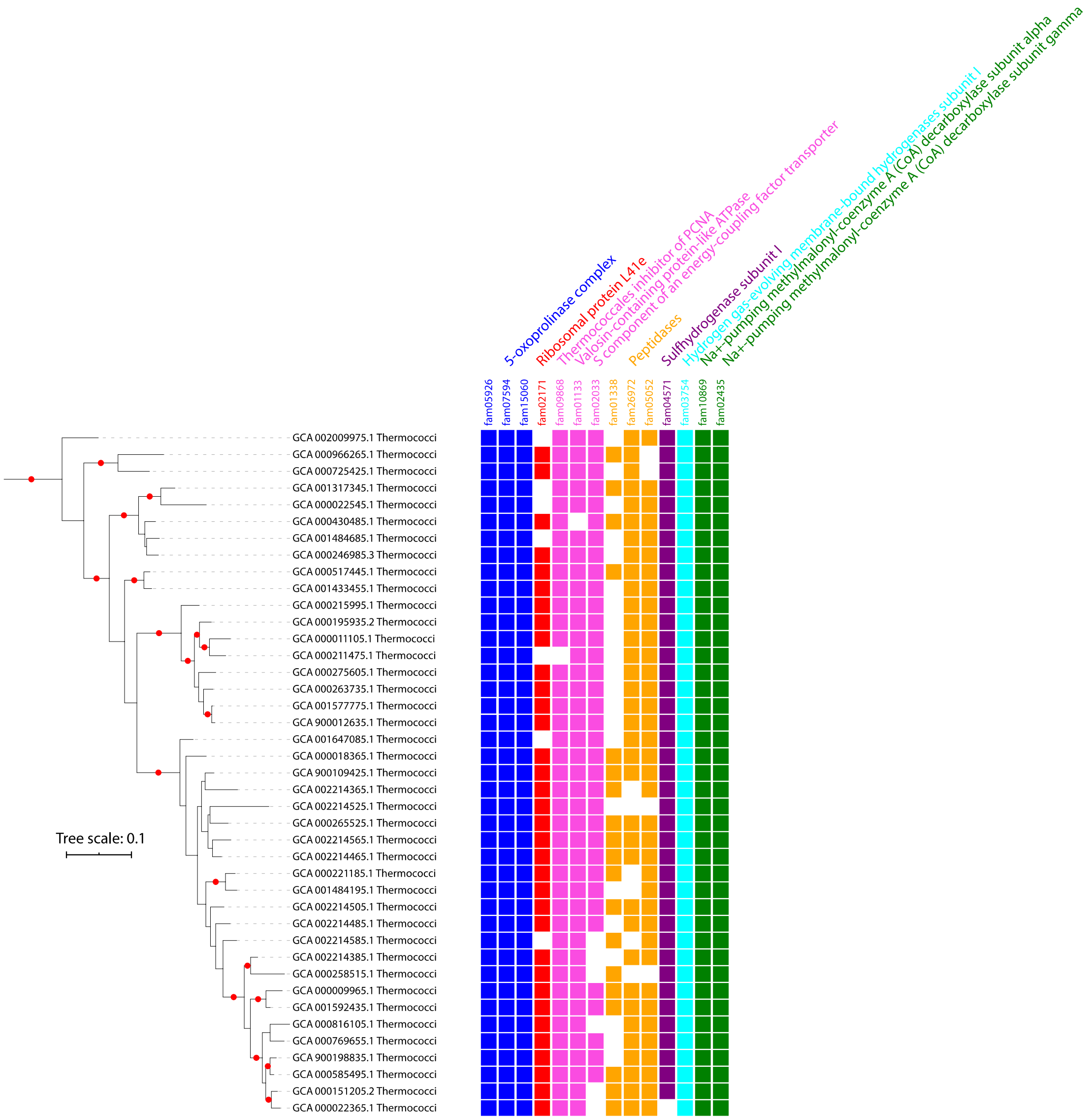

Supplementary Figure S12. Presence and absence of 14 families of module 8 in genomes of Thermococci. Scale bar indicates the average substitutions per site.

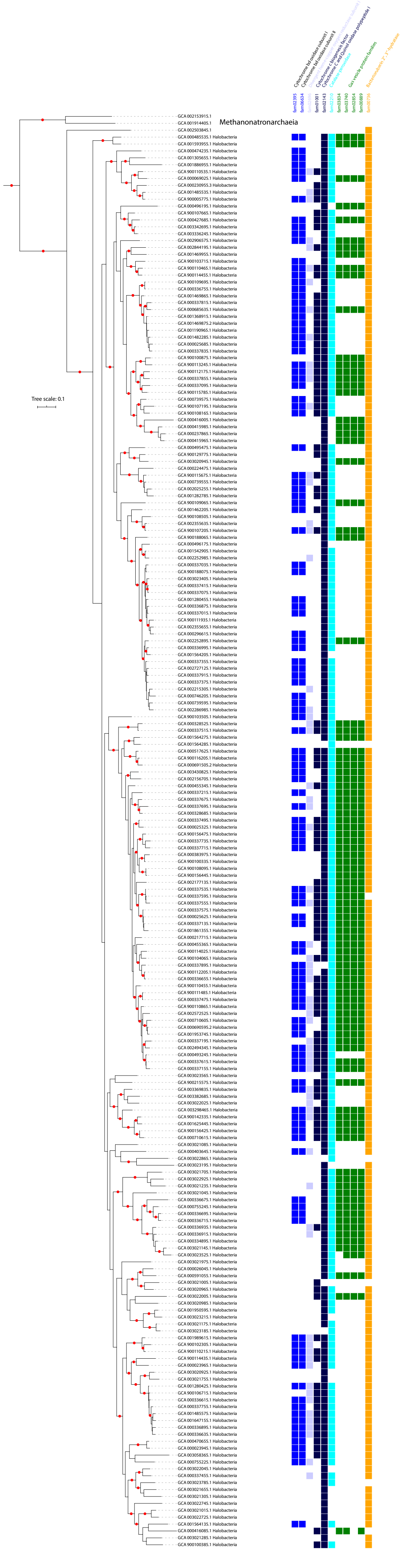
Cytochrome bd oxidase subunit I
Cytochrome bd oxidase subunit II
Divergent heme-copper oxygen reductase subunit I
Cytochrome c biogenesis factor
Cytochrome C and Quinol oxidase polypeptide I
Catalase-peroxidase
Gas vesicle protein families
Bacterioruberin 2'', 3''-hydratase
fam02395
fam06634
fam02696
fam01001
fam02143
fam02210
fam03834
fam03740
fam02854
fam00889
fam00736
Methanonatronarchaeia
Tree scale: 0.1

Supplementary Figure S13. Presence and absence of 11 families of modules 13 and 108 in genomes of Halobacteria. Scale bar indicates the average substitutions per site.

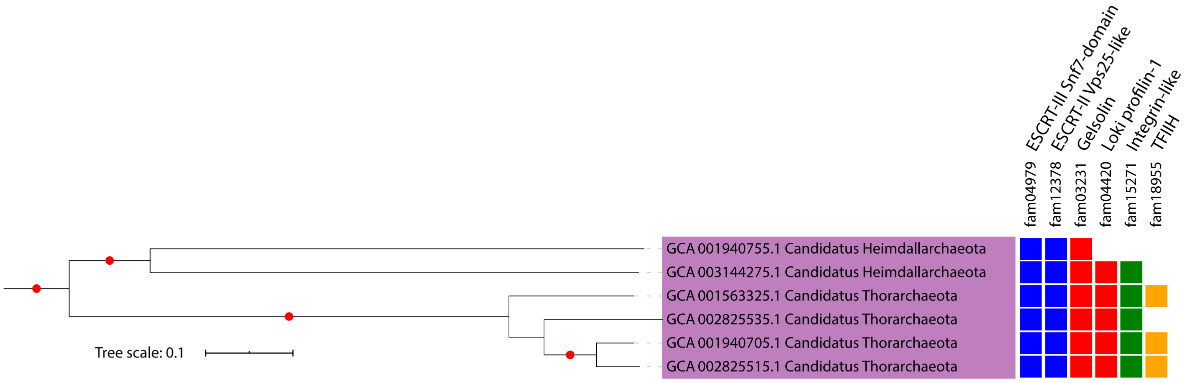

Supplementary Figure S14. Presence and absence of 6 families of module 48 in genomes of Asgard. Scale bar indicates the average substitutions per site.

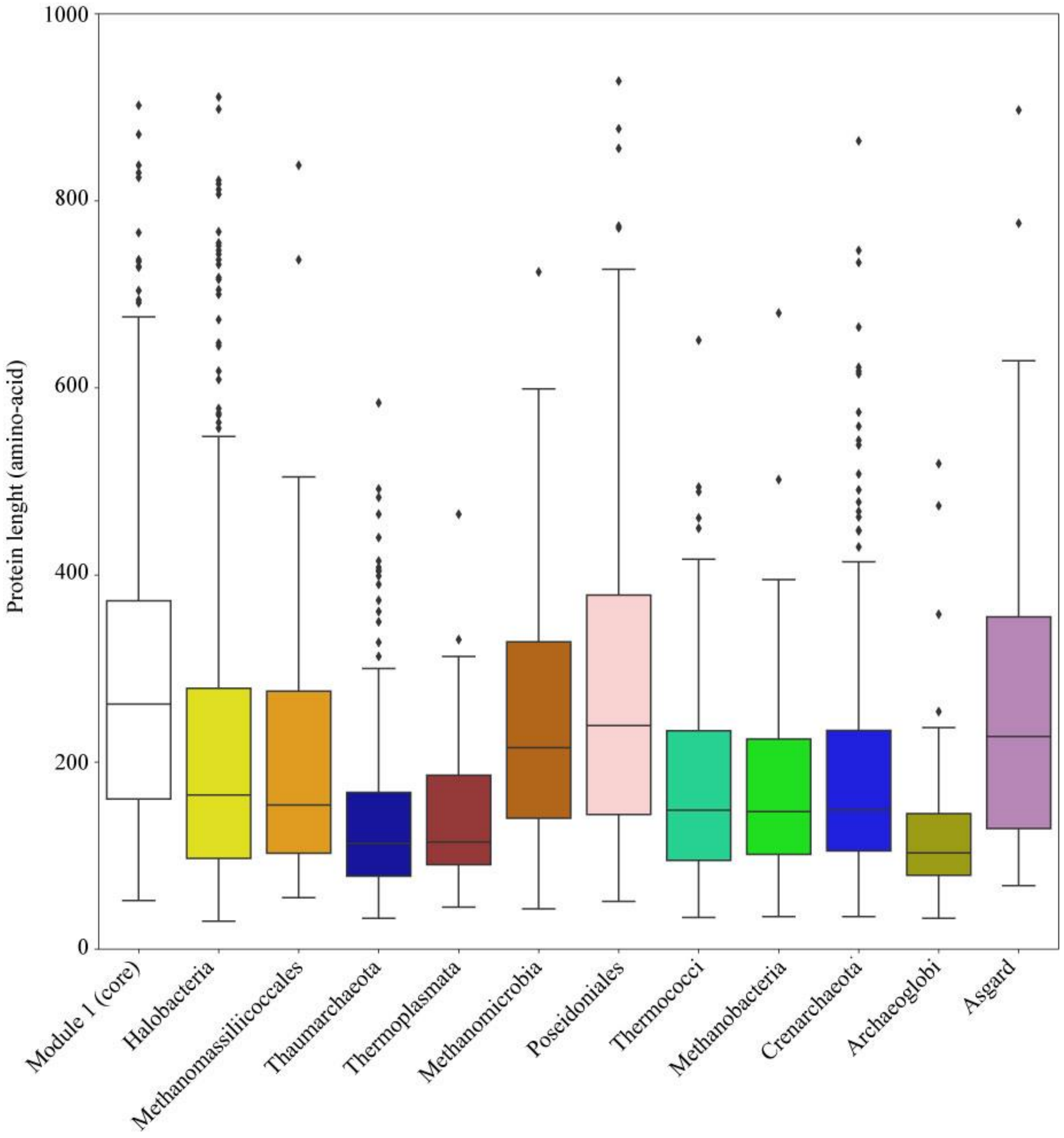

Supplementary Figure S15. The length distribution of hypothetical proteins (in amino acid).
