# Supplementary material for: Conserved and lineage-specific hypothetical proteins may have played a central role in the rise and diversification of major archaeal groups
Source: BMC Biol. 2022 Jul 5;20:154. doi: 10.1186/s12915-022-01348-6 (PMC9258230; doi:10.1186/s12915-022-01348-6)
Supplement: Supplementary file 3 — Additional file 3. [file 12915_2022_1348_MOESM3_ESM.docx]

Additional File 3: Supplementary information

for

Conserved and lineage-specific hypothetical proteins may have played a central role in the rise and diversification of major archaeal groups

Raphaël Méheust^+,1,2,3^, Cindy J. Castelle^1,4^, Alexander L. Jaffe^5^ and Jillian F. Banfield^+,1,2,4,6^

^+^Corresponding authors: jbanfield@berkeley.edu, raphael.meheust@genoscope.cns.fr

^1^Department of Earth and Planetary Science, University of California, Berkeley, CA, USA

^2^Innovative Genomics Institute, University of California, Berkeley, CA, USA

^3^LABGeM, Génomique Métabolique, Genoscope, Institut François Jacob, CEA, Evry, France

^4^Chan Zuckerberg Biohub, San Francisco, CA, USA

^5^Department of Plant and Microbial Biology, University of California, Berkeley, CA, USA

^6^Department of Environmental Science, Policy, and Management, University of California, Berkeley, CA, USA

**Do methanogens cluster together, despite their phylogenetic diversity?** (Full analysis)

To test whether we could detect expected patterns of protein family distribution across lineages using our approach, we examined the distribution of genes involved in methanogenesis, including the alpha subunit *mcrA*, a key gene in methane production [[32]](https://paperpile.com/c/HolgZU/vctj). The mcrA protein family (fam05485) was identified in module 65, which comprises 128 protein families and is highly enriched in Methanomicrobia, Methanobacteria and Methanomassiliicoccales (**Additional file 2: Figure S8**). Along with the *mcrA* gene, all the other subunits (BCDG) of methyl–coenzyme M reductase (Mcr) were identified in module 65 (fam02993, fam03716, fam15638 and fam04416). Five subunits of the methyl-tetrahydromethanopterin (methyl-H4MPT): coenzyme M methyltransferase (Mtr) (BCDEF) were also found (fam06037, fam06163, fam06462, fam06013 and fam02119 respectively) although the *Mtr* genes were absent in *Methanomassiliicoccales* [[33]](https://paperpile.com/c/HolgZU/FFOB) (**Additional file 1: Table S3**). Using HMM-HMM comparison method against the eggNOG database [[26]](https://paperpile.com/c/HolgZU/qYRm) (see Methods), we also detected five hypothetical conserved protein families that are associated with the core proteins of methanogenesis (fam02372, fam05394, fam06062, fam06600 and fam12037) [[33]](https://paperpile.com/c/HolgZU/FFOB) (**Additional file 1: Table S4**). The occurrence between the Mcr subunits and the five methanogenesis markers of unknown functions is striking (**Additional file 2: Figure S8**). Further, genes for transport of iron, magnesium, cobalt and nickel and for synthesis of key cofactors that are required for methanogens growth were also found in the module 65. We identified two other modules enriched in subunits of the energy-converting hydrogenase A (fam10726, fam14360, fam17633, fam06266, fam13666, fam08995, fam03063, fam02679, fam22457 and fam06367) and B (fam06098, fam06875 and fam32156) (module 129) and in enzymes for the utilization of methylamine (fam02336 and fam03937), dimethylamine (fam03076 and fam05873), and trimethylamine (fam04092 and fam21299) as substrates for methanogenesis [[35]](https://paperpile.com/c/HolgZU/my6w) (module 184). Methylamine, dimethylamine and trimethylamine are in two distinct families instead of one single family due to mispredictions of the coding sequences (each gene was split in two consecutive genes by the gene prediction software Prodigal). We found two families of methanol---5-hydroxybenzimidazolylcobamide Co-methyltransferase in two distinct families (fam05405 and fam04064) in modules 184 and 72 that are specific to the Methanomassiliicoccales (**Additional file 2: Figure S8** and **Additional file 1: Table S3**). When the distribution of the signature families is rendered on the phylogenetic tree of Archaea the correspondence between families, modules and annotations is apparent (**Additional file 2: Figure S8**).

We also recovered mcr subunits in lineages that are not considered as canonical methanogenic lineages [[36]](https://paperpile.com/c/HolgZU/IFWj). These include two genomes of Bathyarchaeota related to BA1 and BA2 (GCA_002509245.1 and GCA_001399805.1) [[37]](https://paperpile.com/c/HolgZU/GDgh), and one Archaeoglobi genome related to JdFR-42 (GCA_002010305) [[38, 39]](https://paperpile.com/c/HolgZU/wP8a+YO5s). These genomes have been described as having divergent MCR genes. It is reassuring that our method is sensitive enough to recover distant homology (**Additional file 2: Figure S8**).

**Functions specific to Poseidoniales.** (Full analysis)

Modules 32 and 71, encompassing 199 families, were consistently associated with genomes of Poseidoniales archaea. Recent comparative genomic analysis of 250 Poseidoniales genomes revealed the ecological roles of these archaea in protein and saccharide degradation [[43]](https://paperpile.com/c/HolgZU/GWH4). Protein degrading enzymes (several different classes of peptidases and one oligotransporter) found in modules 32 and 71, include some previously linked to Poseidoniales archaea (fam05120, fam05272 and fam01092) [[43]](https://paperpile.com/c/HolgZU/GWH4). We also identified two new Poseidoniales-specific families of well-conserved peptidases that are seemingly unique to Poseidoniales (peptidase M17; PF00883; fam03211 (Module 32) and peptidase M6; PF05547 fam00840 (Module 71)). As reported by Tully [[43]](https://paperpile.com/c/HolgZU/GWH4), peptidase S15 (PF02129; fam03321) and peptidase M60-like (PF13402; fam05454) have a narrow distribution within Poseidoniales so were assigned to another module (module_738). Interestingly, we identified modules specific to Poseidoniales subgroup IIa (module_135, containing 99 families) and Poseidoniales subgroup IIb (module_45, containing 39 families). Both modules contain 4 protein families with calcium-binding domains (fam02857, fam00852 and fam02101 in module 135 and fam01838 in module 45) (**Additional file 2: Figure S9**). These proteins may be involved in signaling and regulation of protein-protein interactions in the cell [[44]](https://paperpile.com/c/HolgZU/unM7).

**Functions specific to the six Asgard genomes** (Full analysis).

The module 48 contains 42 families that are specific and conserved in the six genomes of the superphylum Asgard (four genomes of Thorarchaeota and two genomes of Heimdallarchaeota). Of these, 33 lack both KEGG and PFAM functional predictions (**Additional file 1: Table S3**). The Asgard archaea, which affiliate with eukaryotes in the tree of life [[7]](https://paperpile.com/c/HolgZU/bwyb), encode proteins that they only share with eukaryotes [[68]](https://paperpile.com/c/HolgZU/vgg3). We detected six eukaryotic signature protein families (ESPs) in module 48 (**Additional file 2: Figure S14**). These ESPs include the ESCRT-III Snf7-domain family (fam04979) and the ESCRT-II Vps25-like family (fam12378). These two families are part of the ESCRT system and the genes are found in the same genomic region in the Asgard genomes [[8]](https://paperpile.com/c/HolgZU/Je5H).

Three cytoskeleton-related families were found in module 48. The two first families are the gelsonin-domain protein family (fam03231) [[8]](https://paperpile.com/c/HolgZU/Je5H) and fam04420 that matches with the PDB sequence of the Loki profilin-1 crystal structure (accession: 5yee) [[69]](https://paperpile.com/c/HolgZU/zURD). Interestingly, the third cytoskeleton-related family (fam15271) shows similarity with the integrin beta 4. These proteins do not share sequence similarity with the integrin repeat-containing ESPs recently identified in Asgard genomes [[70]](https://paperpile.com/c/HolgZU/OTOn) and may represent a new ESP. The genes of fam15271 are always located next to tubulin genes (fam00241) in the five Asgard genomes (**Figure 3A and Additional file 1: Table S8**). This is particularly interesting as recent studies have shed light on the crosstalk between integrin and the microtubule cytoskeleton [[71]](https://paperpile.com/c/HolgZU/xmdR).

Finally, one family in module 48 (fam18955) is annotated as the DNA excision repair protein ERCC-3 in three Asgard genomes and three Theionarchaea genomes. The genes neighboring the genes of fam18955 differ between the two lineages (**Figure 3B** and **Additional file 1: Table S8**) and the three Asgard sequences only share between 20 and 23% protein identity with the three Theionarchaea sequences. These differences may indicate two distinct functions for this family. Fam18955 shows distant homology with the protein RAD25 of *Saccharomyces cerevisiae*. RAD25 is a DNA helicase required for DNA repair and RNA polymerase II transcription in *S. cerevisiae* [[72]](https://paperpile.com/c/HolgZU/ixYf). RAD25 is also one of the six subunits of the transcription factor IIH (TFIIH) in *S. cerevisiae* [[73]](https://paperpile.com/c/HolgZU/WJ46). Consistent with the role of RAD25 in *S. cerevisiae*, the genes of family18955 is found next to replication factor C small subunit genes in the three Asgard genomes (**Figure 3** and **Additional file 1: Table S8**).
